# New peptide derived antimalaria and antimicrobial agents bearing sulphonamide moiety

**DOI:** 10.1080/14756366.2019.1651313

**Published:** 2019-08-08

**Authors:** D. I. Ugwuja, U. C. Okoro, S. S. Soman, R. Soni, S. N. Okafor, D. I. Ugwu

**Affiliations:** a Department of Chemical Sciences, Federal University, Wukari, Nigeria;; b Department of Pure and Industrial Chemistry, University of Nigeria, Nsukka, Nigeria;; c Department of Chemistry, The Maharaja Sayajirao University of Baroda, Vadodara, India;; d Department of Pharmaceutical and Medicinal Chemistry, University of Nigeria, Nsukka, Nigeria

**Keywords:** Dipeptide carboxamide, benzensulphonamoyl propanamide, synthesis, antimalarial, antimicrobial, molecular docking

## Abstract

Fourteen novel dipeptide carboxamide derivatives bearing benzensulphonamoyl propanamide were synthesized and characterized using ^1^H NMR, ^13^C NMR, FTIR and MS spectroscopic techniques. *In vivo* antimalarial and *in vitro* antimicrobial studies were carried out on these synthesized compounds. Molecular docking, haematological analysis, liver and kidney function tests were also evaluated to assess the effect of the compounds on the organs. At 200 mg/kg body weight, **7i** inhibited the multiplication of the parasite by 81.38% on day 12 of post-treatment exposure. This was comparable to the 82.34% reduction with artemisinin. The minimum inhibitory concentration (MIC) in µM ranged from 0.03 to 2.34 with **7h** having MIC of 0.03 µM against *Plasmodium falciparium.* The *in vitro* antibacterial activity of the compounds against some clinically isolated bacteria strains showed varied activities with some of the new compounds showing better activities against the bacteria and the fungi more than the reference drug ciprofloxacin and fluconazole.

## 1. Introduction

Malaria stands among one of the terrible diseases that have been known for more than 4000 years^1^. Malaria is an infectious disease caused by protozoa of the genus Plasmodium and is transmitted to humans by the Anopheles mosquito. Since this malady is mainly present in tropical regions and primarily affects poor people in developing countries, its occurrence is often associated with socioeconomic problems^2^. At present, artemisinin and its combinational therapy is the most appropriate drug therapy for malaria in the malaria endemic region, but due to spread of artemisinin resistance in five countries, Cambodia, the Lao People’s Democratic Republic, Myanmar, Thailand and Viet Nam^3^, urges the need for the development of new antimalaria. To this concern, lots of antimalaria and other therapeutic approaches are in the pipeline to impede the growth of this disease, including antimalaria peptides^4^. Peptides are among the most versatile bioactive molecules and play crucial roles in the human body and other organism^5^. Because of their good solubility, permeability and bioavailability, many peptide hormones and analogues of short peptides exert their action by binding to membrane receptors^6^^,^^7^ or possess signalling and regulatory functions. There are peptides (cell-penetrating as well as non-penetrating) that can be conjugated to a cargo to deliver it to a specific organ,^8^ However, despite the advantages of the antimalaria peptide of having high target selectivity that reduces the usual side effects of small and low molecular weight drug, peptides mostly offer various challenges before it can be used as a therapeutic drug.^9^

Sulfonamides are widely used in medicinal chemistry because of their low cost, low toxicity and excellent biological activity. For example, sulfadoxine, sulfadiazine and sulfalene are effective malaria drugs that possess sulfonamide groups attached to a heterocyclic ring.

Many drugs possess the functionality of primary sulfonamides^10^ defined by (RSO_2_NH_2_) compounds. Secondary or tertiary sulfonamides (R-SO_2_NR_1_R_2_) are defined by the single or double N-alkyl or N-aryl/heteroaryl substitution of the primary sulfonamide, respectively. As there is a continuous need of molecule to be introduced in the treatment of diseases, the use of substituted sulfonamide is a valid approach to expand the number of new lead compound to be further developed. Such results can be achieved as there is easy accessibility of derivatives, low toxicity profile, and possibility to manipulate chemical structures under mild conditions^11^.

Krungkrai and coworkers reported a library of aromatic/heteroaromatic sulfonamides with diverse scaffolds and assayed these compounds for the inhibition of carbonic anhydrase from *Plasmodium falciparum,* (pfCA)^12^^,^^13^. The literature describes several other sulfonamides with antimalarial activity^14–20^. Some of these studies involve the inhibition of folate metabolic enzymes that are crucial for the growth of the malaria parasite. Some of these enzymes are absent in humans and are therefore potential targets for malarial chemotherapy. In general, the sulfonamide functional group has an impressive effectiveness and assuredness history in medicine. It usually presents interesting pharmacokinetic properties being well absorbed by several routes of administration, presenting good penetration into tissues and fluids, and being easily metabolized^21^^,^^22^. In particular, in the last decade, it has been widely employed in the treatment of malaria^23–25^.

The reported emergence of insecticide-resistant mosquitoes and artemisinin-resistant malaria parasite calls for concerted effort in the development of new antimalarial drugs. Malaria is often times accompanied with microbial infections leading to complications; this necessitates the development of new antimalarial drugs with additional antimicrobial activity. To respond to the problems associated with current antimalarial drugs, a design of compounds containing sulfonamide, carboxamide and peptide functionality is ideal given their reported individual antimalarial properties. The synergistic activity arising from the successful combinations of these functionalities is exploited in this research. We report herein the synthesis of short peptides containing sulfonamide and carboxamide functionalities with interesting antimalarial and antimicrobial activity.

## 2. Material and methods

Reagent-grade chemicals and solvents were purchased from commercial supplier and used after purification. Thin-layer chromatography (TLC) was performed on silica-gel F254 plates (Merck). Merck silica gel (60–120 mesh) was used for column chromatographic purification. All reactions were carried out in a nitrogen atmosphere. Melting points are uncorrected and were measured in open capillary tubes, using a Rolex melting-point apparatus. IR spectra were recorded as KBr pellets on a Perkin-Elmer RX 1 spectrometer and the wave numbers are reported in cm^−1^. ^1^H NMR and ^13^C NMR spectral data were recorded on Advance Bruker 400 spectrometer (^1^H 400 MHz/^13^C 100 MHz) with DMSO-d_6_ as solvent and tetramethylsilane (TMS) as internal standard and reported in *δ* (ppm). *J* values are in hertz (Hz). Mass spectra were determined by High Resolution Mass Specter (HRMS).

### 2.1. General procedure for the synthesis of substituted benzene sulphonamoyl Alkanamides (3a and 3b)^26^

Sodium carbonate (Na_2_CO3, 1.590 g, 15 mmol) was added to asolution of amino acids (**2**,12.5 mmol) in water (15 mL) with continuous stirring until all the solutes had dissolved. The solution was cooled to5 °C and the appropriate benzenesulphonyl chloride (**1a–b**, 15 mmol) was added in four portions over a period of 1 h. The slurry was further stirred at room temperature for about 4 h. The progress of the reaction was monitored using TLC (MeOH/DCM,1:9). Upon completion, the mixture was acidified using 20% aqueous hydrochloric acid to pH 2. The crystals were filtered via suction and washed with pH 2.2 buffer. The pure products (**3a–b**) were dried over self-indicating fused silica gel in a desiccator.

### 2.2. General procedure of preparing compound 6a–6e^27^

A mixture of substituted analytical grade of Boc-glycine (**4**, 0.45 g, 1.84 mmol), 1-ethyl-3–(3-dimethyl aminopropyl carbodiimide hydrochloride (0.53 g, 2.76Mmol) EDC, 1-hydroxybenzotriazole (0.248, 1.84Mmol) HOBT, Triethylamine (TEA), amines (0.515 g 1.84Mmol) in dichloromethane (50 ml) DCM was stirred at room temperature for sixteen (16) h. The reaction was monitored using TLC. On completion of the reaction, it was washed with water (2 × 20 ml), brine (1 × 10 ml), dried over anhydrous sodium sulfate and the solvent was evaporated under reduced pressure to give the crude product(**5a–g**) which was then purified by column chromatography using silica gel. The products (**5a–g**) were then deprotected by addition of 10% TFA in DCM and allow to stir for more than 1 h and been monitored with TLC. When the reaction is completed, the solvent is evaporated, and the products (**6a–g**) were used directly for the next stage of reaction.

### 2.3. General procedure of preparing 7a–7n

A mixture of 2-[phenylsulfonyl)amido]propanioc acid (**3a–b**, 1.84 mmol), 1-ethyl-3–(3-dimethyl aminopropyl carbodiimide hydrochloride (0.53 g, 2.76 Mmol) EDC, 1-hydroxybenzotriazole (0.248, 1.84 Mmol) HOBT, Triethylamine (TEA), deprotected boc-glycine amine (**6a–g**, 1.84 Mmol) in dichloromethane (50 ml) DCM was stirred at room temperature for 16 h. The reaction was monitored using TLC. On completion of the reaction, it was washed with water (2 × 20ml), brine (1 × 10ml), dried over anhydrous sodium sulfate and the solvent was evapourated under reduced pressure to give the crude product **(7a–n)** which was then purified by column chromatography using silica gel.

#### 2.3.1. Compounds 7a–7n

##### 2.3.1.1. N-(2-((3-chlorophenyl)amino)-2-oxoethyl)-2-(phenylsulfonamido)propanamide(7a)

Yield 71% mp 172–174 °C FTIR(KBrcm^−1^), 3348.6(NH), 3003.9(C-H aro), 2985.4, 2934.6 (C-H), 1665.1, 1593.1(C = O), 1526.7, 1478.7, 1446.4(C = C), 1377.5 (SO_2_), 1168.4 (SO_2_NH), 758.0 (C-Cl) ^1^HNMR (DMSO-d_6_ 400 MHz): 8.2(s, 1H), 8.1–8.1(d, 1H), 7.8–7.7(d, 2H), 7.6–7.5(m, 1H), 7.5–7.5(m, 3H), 7.4–7.4(d, *J* = 8 Hz), 7.3–7.3(m, 4H), 7.1–7.0(d, *J* = 7.6 Hz, 1H), 3.8–3.7(m, 1H), 1.0–1.0(D, *J* = 6.8 Hz, 3H) ^13^CNMR (DMSO-d_6_ 100 MHz); 19.0, 42.9, 52.2 (3 aliphatic carbon), 117.8, 118.8, 123.4, 127.0, 129.4, 131.0, 132.8, 133.5, 140.6, 141.3 (aromatic carbon), 168.2, 172.1 (carbonyl carbon). HRMS-ESI found value is (*m/z*): 395.02, M^+^, calculated C_17_H_19_ClN_3_O_4_S value is 395.07.

##### 2.3.1.2. N-(2-((4-chlorophenyl)amido)-2-oxoethyl)-2-oxoethyl)-2-(phenylsulfonamido)propanamide(7b)

Yield 80%, mp 140–142 °C. FTIR(KBrcm^−1^), 3325.3, 3270.8(2NH), 3066.7(C-H Ar), 2986.8, 2933.2(C-H), 1646.5, 1646.5(C = O), 1598.0, 1530.8, 1492.5, 1448.0 (C = C), 1400.0, 1328 (2 SO_2_), 1166.9 (SO_2_NH), 785.8(C-Cl) ^1^HNMR(DMSO-d_6_ 400 MHz): 1.0–1.0(d, *J* = 7.2 Hz, 3H), 3.7–3.8(m, 1H), 7.3–7.3(m, 3H), 7.5–7.5(m, 6H), 7.6–7.6(d, *J* = 2H), 7.6–7.6(d, *J* = 1H), 7.7–7.8(m, 1H), 8.0(s, 1H), 8.2–8.2(m, 1H), 10.0(s, 1H) ^13^CNMR(DMSO-d_6_ 100 MHz): 19.0, 43.0, 52.3(aliphatic carbon), 121.0, 124.6, 125.9, 126.8, 127.0, 128.6, 129.4, 129.5, 132.2, 132.8, 138.0, 141.3, 166.4 (aromatic carbon), 167.9, 172.1 (carbonyl carbon). HRMS-ESI found value is (*m/z*): 395.02, M^+^, calculated C_17_H_19_ClN_3_O_4_S value is 395.07.

##### 2.3.1.3. N-(2-((3-fluorophenyl)amido)-2-oxoethyl)-2-oxoethyl)-2-(phenylsulfonamido)propanamide(7c)

Yield 66%, mp 125–127 °C. FTIR(KBrcm^−1^),3332.7(NH), 3088.7, 2980.0(C-H Ar), 2939.0, 2857.0(C-H), 1669.7, 1607.9(C = 0), 1529.0, 1491.5, 1445,3(C = C), 1374.1, 1320.4(2SO_2_), 1166.9(SO_2_NH), 686.9(C-Fl) ^1^HNMR(DMSO-d_6_ 400 MHz): 1.0–1.0(d, *J* = 6.8 Hz. 3H), 3.7–3.8(m,1H), 6.6–6.8(m, 1H), 7.2–7.3(m, 2H), 7.5–7.6(m, 4H), 7.8–7.8(d, *J* = 7.2 Hz, 2H), 8.0–8.0(d, *J* = 8.4 Hz, 3H), 8.5–8.5(d, *J* = 7.6 Hz, 1H), 8.2–8.2(m, 1H), 10.0(s, 1H) ^13^CNMR(DMSO-d_6_ 100 MHz): 19.0, 45.0, 52.3(aliphatic carbon), 106.4, 110.3, 115.2, 127.0, 129.4, 130.9, 132.8, 140.8, 141.3, 161.4, 163.7 (aromatic carbon), 168.1, 172.1 (carbonyl carbon). HRMS-ESI found value is (*m/z*): 378.09, M-H, calculated C_17_H_19_FN_3_O_4_S value is 379.10

##### 2.3.1.4. N-(2-((3-florophenyl)amino)-2-oxoethyl)-2-(phenylsulfonamido)propanamide(7d)

Yield 77% mp 168–170 °C FTIR(KBrcm^−1^), 3333.01(NH), 3087(C-H Ar), 2979.2, 2939.5(C-H), 1695.5, 1670.5 (C = O), 1609.9, 1529.5, 1492.8, 1445.2(C = C), 1375.4(SO_2_), 687.6(C-F) ^1^HNMR (DMSO-d_6_): 10.1(s, 1H), 8.2–8.2(triplet. 1H), 7.8–7.7(m, 2H), 7.6–7.5 (m, 3H), 7.5–7.5(m, 4H), 7.4–7.2(m, 8 H), 6.8–6.8(ddd, 1H), 3.8–3.7(m,1H), 1.0(d, *J* = 7.2 Hz 3H) ^13^CNMR (DMSO-d_6_ 1OOMHz):19.0, 42.9, 52.3 (aliphatic carbon), 106.1, 106.4, 110.1, 110.3, 115.2, 126.9, 129.5, 130.9, 132.9, 140.8, 140.9, 141.1, 161.3, 163.7, aromatic carbon, 168.16, 172.25 carbonyl carbon. HRMS-ESI found value is (*m/z*): 378.09, M-H, calculated C_17_H_19_FN_3_O_4_S value is 379.10.

##### 2.3.1.5. N-(2-oxo-2)-(p-tolylamino)ethyl)-2-(phenylsulfonamido)propanamide(7e)

Yield 73% mp 160–162 °C. FTIR(KBrcm^−1^),3354.5, 3290.0(NH), 3059.2(C-H aro), 2968.5, 2928.9(C-H), 1653.1, 1602.3(C = O), 1534.3, 1447.1(C = C), 1378.7(SO_2_), 171.1(SO_2_NH) 1HNMR (DMSO-d_6_ 400 MHz): 9.7(s, 1H), 8.2–8.2(m, 1H), 7.8–7.7(m, 2H), 7.6–7.5(m, 3H), 7.5–7.5(m, 4H), 7.4–7.4(d, *J* = 8.2 Hz, 1H), 2.2(m, 1H), 1.0–1.04(d, *J* = 7.2 Hz, 3H) 13NMR (DMSO-d_6_ 100 MHz): 19.0, 20.0, 42.9, 52.2 (aliphatic carbon), 119.4, 127.0, 129.5, 129.6, 132.6, 132.9, 136.7, 141.2,(aromatic carbon), 167.4, 172.0 (carbonyl carbon). HRMS-ESI found value is (*m/z*): 376.13, M + H, calculated value is 375.13.

##### 2.3.1.6. N-(2-oxo-2-(phenylamino)ethyl)-2-(phenylsulfonamido)propanamide(7f)

Yield 68%, mp 140–142 °C FTIR(KBrcm^−1^), 3340.6(NH), 3084.7(C-H Ar), 2979.7,(C-H), 1693.3, 1664.8(C = O), 1596.1, 1528.3, 1499.7, 1480.2(C = C), 1375.8(S0_2_), 1167.1(SO_2_NH) ^1^HNMR (DMSO-d_6_, 400 MHz):1.0–1.1(d, *J* = 32 Hz, 3H), 2.4(s, 1H), 3.0(s, 3H), 3.7–4.0(m, 2H), 7.0(s,1H), 7.2(s, 2H), 7.5–7.8(m, 3H), 8.1(s, 1H), 8.2(s, 2H), 9.8–9.8(m, 2H) ^13^CNMR (DMSO-d_6_, 100 MHz): 19.0, 43.0, 52.3(aliphatic carbon), 119.4, 119.5, 123.7, 123.9, 126.8, 127.0, 129.2, 129.4, 132.8, 139.2, 141.2(aromatic amine) 167.7, 172.1(carbonyl carbon). HRMS-ESI found value is (*m/z*): 361.1, M^+^, calculated C_17_H_19_N_3_O_4_S value is 361.10.

##### 2.3.1.6. N-(2-(naphthalene-2-ylamino)-2-oxoethyl)-2-(phenylsulfonamido)propanamide(7g)

Yield 70%, mp 170–172 °C FTIR(KBrcm^−1^), 3346.7, 3253.6(NH), 3066.9(C-H Ar), 2977.6, 2935.9(C-H), 1649.1, 1598.6(C = O), 1529.8, 1502.3, 1461.7, 1447.5(C = C), 1375.3(SO_2_), 1170.9(SO_2_NH). ^1^HNMR (DMSO-d_6_, 400 MHz: 1.0(d, *J* = 5.2 Hz, 3H), 4.0–3.9(M, 1H), 4.0(br s 2H), 7.6–7.4(M, 8 H), 7.8–7.7(m, 3H), 7.9–7.9(m, 1 H) , 8.1–8.0(m, 2H), 8.3(S, 1H). ^13^NMR(DMSO-d_6_ 100 MHz): 19.0, 43.1, 52.3 (aliphatic carbon), 121.9, 123.1, 125.8, 126.0, 126.3, 126.5, 127.0, 128.1, 128.5, 129.3, 129.4, 129.6, 132.8, 133.5, 134.1, 141.3(aromatic carbon), 168.5, 172.2 carbonyl carbon. HRMS-ESI found value is (*m/z*): 412.1, M + H, calculated C_21_H_21_N_3_O_4_S value is 411.1.

##### 2.3.1.7. N-(2-((3-chlorophenyl)amino)-2-oxoethyl)-2–(4-methylphenylsulfonamido)propanamide(7h)

Yield 66% mp 120–122 °C FTIR(KBrcm^−1^), 3361.4, 3324.9, (2NH), 3078.2, 2979.0(C-H Ar), 2939.3, 2857.8(C-H), 1857.0, 1667.3(2 C = O), 1593.3, 1526.6, 1478.87, 1446.5(C = C), 1376.9, (SO_2_) 1169.2(SO_2_NH), 1780.2(C-Cl) ^1^HNMR (DMSO-d_6_ 400 MHz): 1.0–1.0(d, *J* = 6.8 Hz, 3H), 2.3(s, 4H), 3.8(s, 3H), 7.0–7.1(d, *J* = 6.8 Hz, 1H), 7.3–7.3(d, 7.2 Hz, 4H), 7.4–7.4(d, *J* = 7.6 Hz, 2H), 7.6–7.6(m, 3H), 7.7(s, 1H), 7.9–7.9(m, 1H), 8.2(s, 1H), 10.1(s, 1H). ^13^CNMR(DMSO-d_6_ 100 MHz); 19.0, 21.4, 43.0, 52.3 (aliphatic carbon), 117.8, 118.9, 123.4, 127.0, 129.9, 130.9, 133.5, 138.4, 140.6, 143.0(aromatic carbon), 168.1, 172.2(carbonyl carbon). HRMS-ESI found value is (*m/z*): 432.14, M + Na, calculated C_18_H_21_ClN_3_O_4_S value is 409.09.

##### 2.3.1.8. N-(2-((3-chlorophenyl)amino)-2-oxoethyl)-2–(4-methylphenylsulfonamido)propanamide(7i)

Yield 82%, mp 160–162 °C FTIR(KBrcm^−1^), 3344.0, 3250.3(2NH), 3115.4, 3067.5(C-H Ar), 2985.1, 2920.8(C-H), 1675.1, 1648.0(2 C = O), 1596.1, 1526.5, 1493.12, 1449.3, 1430.3(C = C), 1332.8, (SO_2_), 1164.(SO_2_NH), 711.7(C-Cl). ^1^HNMR(DMSO-d_6_, 400 MHz) 1.0(s, 3H), 3.7–3.8(d, *J* = 8H, 2H), 7.3(s, 1H), 7.5–7.5(d, *J* = 4 Hz 2H), 7.7(s, 1H), 8.1–8.2(M 2H, 10.01(S, 1H) ^13^CNMR(DMSO-d_6_, 100 MHz); 19.3, 42.9, 52.2,(aliphatic carbon) 121.0, 127.3, 128.9, 129.1, 129.5, 137.6, 138, 140.3,(aromatic carbon) 167.8, 171.9(carbonyl carbon). HRMS-ESI found value is (*m/z*): 432.1, M + Na, calculated C_18_H_21_ClN_3_O_4_S value is 409.1.

##### 2.3.1.9. N-(2-((3-fluorophenyl)amino)-2-oxoethyl)-2–(4-methylphenylsulfonamido)propanamide(7j)

Yield 75% mp 122–124 °C FTIR(KBrcm^−1^): 3333.5(NH), 3089.8(C-H Ar), 2978.7, 2938.1(C-H), 1696.7, 1665.0(C = O), 1601.9, 1535.6, 1494.3, 1445.0(C = C), 1322(SO_2_), 1169.5(SO_2_NH). 684.9(C-Fl) ^1^HNMR(DMSO-d_6_, 400 MHz):1.0–1.1(d, *J* = 32 Hz, 3H), 2.3–2.9(m, 1H), 3.77(s, 1H), 6.8(s, 3H), 7.1–7.4(m, 1H), 7.5–7.9(m, 2H), 8.2(s, 1H), 10.1(s, 1H) ^13^CNMR(DMSO-d_6_, 100 MHz):19.0, 21.3, 43.0, 52.3(aliphatic carbon), 106.1, 106.3, 110.1, 110.3, 115.2, 127.0, 129.9, 130.8, 130.9, 138.5, 140.9, 141.0, 143.1, 161.4, 163.8(aromatic carbon), 168.1, 172.2(carbonyl carbon) HRMS-ESI found value is (*m/z*): 393.11, M^+^, calculated C_18_H_21_FN_3_O_4_S value is 393.12.

##### 2.3.1.10. N-(2-((4-fluorophenyl)amino)-2-oxoethyl)-2–(4-methylphenylsulfonamido)propanamide(7k)

Yield 59% mp 176–178 °C FTIR(KBrcm^−1^), 3331.0, 3284.9(NH), 3078.5(C-H Ar), 2985.2, 2929.4(C-H), 1673.4, 1648.7(C = O), 1599.6, 1551.9, 1528.4, 1511.2(C = O), 1376.9(SO_2_), 1163.7(SO_2_NH), 695.6(C-Cl) ^1^HNMR(DMSO-d_6_, 400 MHz): 1.0–1.0(d *J* = 8 Hz 3H),1.1–1.2(m,1H), 1.4–1.4(d, *J* = 4 Hz 2H) 1.5–1.5(d, *J* = 4 Hz, 3H), 2.3–2.3(d, *J* = 7.6 Hz 1H), 3.2(s, 1H), 3.4–3.7(m, 1H), 7.0–7.1(m, 2H), 2.2–7.3(m, 2H), 7.4–7.4(m,2H), 7.5–7.6(m, 1H), 7.9–8.02(d, *J* = 8.8 Hz 2H), 8.2(s,1H) ^13^CNMR(DMSO-d_6_, 100 MHz):19.0, 21.4,42.9, 96.5(aliphatic carbon), 115.7,115.9, 121.1, 121.2, 127.0, 121.9, 135.6, 138.4, 142.9, 143.0, 157.2, 159.6(aromatic carbon)167.6, 172.1(carbonyl carbon). HRMS-ESI found value is (*m/z*): 393.11, M^+^, calculated C_18_H_21_FN_3_O_4_S value is 393.12.

##### 2.3.1.11. 2–(4-methylphenylsulfonamido)-N-(2-oxo-2-(p-tolylamino)ethyl)propanamide(7l)

Yield 82% mp 120–122 °C FTIR(KBrcm^−1^), 3337.1, 3291.8(NH), 3045(C-H Ar), 2982.6, 2919(C-H), 1707.0, 1669.0(C = O), 1599.4, 1532.6, 1448.9, 1430.2(C = C), 1388.2(SO_2_), 1164.3(SO_2_NH) ^1^HNMR(DMSO-d_6_, 400 MHz) 1.0-1.0(d, *J* = 4 Hz, 3H), 2.3(S, 3H), 3.7(S, 3H), 7.3–7.3(d, *J* = 8 Hz, 1H), 7.5–7.6(d, *J* = 8 Hz 2H), 7.6–7.6(d, *J* = 8 Hz 3H), 8.1(s, 1H), 9.9(S, 1H) ^13^CNMR(DMSO-d_6_, 100 MHz); 19.0, 21.4, 43.0, 52.3(aliphatic carbon), 121.0, 127.0, 127.3, 129.1, 129.9, 138.1, 138.4(aromatic carbon), 167.9, 172.2(carbonyl carbon). HRMS-ESI found value is (*m/z*): 388.053, M-H, calculated C_19_H_23_N_3_O_4_S value is 389.14.

##### 2.3.1.12. 2–(4-methylphenylsulfonamido)-N-(2-oxo-2-(phenylamino)ethyl)propanamide(7m)

Yield 74% mp 164–166 °C FTIR(KBrcm^−1^), 3340.7(NH), 3081.5(C-H Ar), 2978.65(C-H), 1693.1, 1664.7,(C-H Ar), 1597.7, 1530.3, 1498.9, 1445.3(C = C), 1324.2(SO_2_), 1167.1(SO_2_NH) ^1^HNMR(DMSO-d_6_ 400 MHz): 1.0–1.0(d, *J* = 8 Hz, 3H), 2.3(s, 3H), 3.3–3.4(d, 3H), 3.7–3.8(m, 3H), 7.0–7.1(m, 1H), 7.2–7.3(m, 4H), 7.5–7.5(d, *J* = 8 Hz, 2H), 7.6–7.7(d, *J* = 8 Hz, 2H), 7.9–7.9(d, *J* = 7.2 Hz 1H), 9.8(s, 1H) ^13^CNMR(DMSO-d_6_ 100 MHz): 19.0, 21.4, 43.05, 52.3(aliphatic carbon), 119.4, 123.7, 127.0, 129.2, 129.2, 129.9, 138.4, 139.2, 143.1(aromatic carbon), 167.7, 172.2(carbonyl carbon) HRMS-ESI found value is (*m/z*): 376.04, M + H, calculated C_18_H_21_N_3_O_4_S value is 375.1.

##### 2.3.1.13. 2–(4-methylphenylsulfonamido)-N-(2-(naphthalene-2-ylamino)-2-oxoethyl)propanamide(7n)

Yield 84%, mp 174–176 °C FTIR(KBrcm^−1^), 3333.3, 3288.0(NH), 3056.6(C-H Ar), 2981.3, 2969.1(C-H), 1743.5 1669.5(C = O), 1598.3, 1534.5, 1461.2, 1448.2 (C = C), 1371 (SO_2_), 1169.9(SO_2_NH) ^1^HNMR (DMSO-d_6_ 400 MHz): 1.07–1.09(s, *J* = 6.8 Hz), 2.34(s, 3H), 3.81–4.0(m, 3H), 7.3–7.3(*J* = 8 Hz, 2H), 7.4–7.5(m, 6H), 7.6—7.7(dd, 4H), 7.9–7.9(d, *J* = 5.2 Hz, 3H), 8.0–8.0(d, *J* = 7.6 Hz), 8.0–8.0(d, *J* = 5.2 Hz, 1H), 8.2(s, 1H), 9.3(s, 1H) ^13^CNMR (DMSO-d_6_ 100 MHz): 19.0, 21.4, 43.1, 52.4 (aromatic carbon), 121.9, 123.1, 125.8, 126.0, 126.3, 127.1, 128.1, 128.5, 129.9, 133.5, 134.1, 138.4, 143.1 (aromatic carbon) 168.5, 172.3 (carbonyl carbon). HRMS-ESI found value is (*m/z*): 426.0, M + H, calculated C_21_H_21_N_3_O_4_S value is 425.1.

### 2.4. Antimicrobial activity

#### 2.4.1. Sensitivity test

*In-vitro* antimicrobial activities of the synthesized compounds were carried out against six bacteria and two fungi of tropical interest. Agar cup diffusion techniques with modification as described by Ugwuja et al.^28^ was used to determine the antimicrobial activity of the compounds. Sensitivity test Muller nutrient agar and plates were seeded with 0.1 ml of overnight culture of micro-organism. The seeded plates were allowed to set after which cups were made in each sector previously drawn from the backside of the bottom plate using marker. Using the sterile pipette, each cup was filled with six drops of their corresponding synthesized compound (2 mg/mL). The solubility solvent was DMF. All the plates were incubated at 37 °C for 24 h and 48 h, respectively, for fungi and bacteria. Zones of clearance round each cup means inhibition and the diameter of such zones were measured. The graph of Inhibition Zone Diameter (IZD) against the log of concentration was plotted for each compound. The anti-log of the intercept on *x*-axis gives the (MIC). The procedure was repeated for fluconazole and ciprofloxacin (standard antifungal and antibacterial agent), and (DMF) (solvent)

#### 2.4.2. Minimum inhibitory concentration (MICs) tests

The (MIC) was determined by further dilution of test sample found to be sensitive against a particular organism. Serial dilutions of the sulfonamides was made to obtain 2.0–0.125 mg/mL. After dilution, the test solutions were added into their corresponding cups previously made in the molten agar starting from the lowest concentration (0.125–2 mg/mL). This was followed by the incubation at the appropriate incubation temperature and time. The resultant inhibition zones diameter (IZD) were measured and values was subtracted from the diameter of the borer (8 mm) to give the IZD. The MIC was also determined by plotting the graph of IZD^2^ against logarithm of concentration for each plate containing a specific compound and micro-organism. The antilogarithm of the intercept on the *x*-axis gives the MIC.

### 2.5. *In vitro* antimalarial assay

The antimalarial activities of the new carboxamides were determined by their inhibition of parasite growth using chloroquine-resistant strain of *P. falciparum*. Effects of inhibitors on parasite development were determined as follows. Sorbitol synchronized, 0.1% parasitemia, ring stage *P. falciparum* strain W2 parasites were cultured under the atmosphere of 3% O_2_, 6% CO_2_ and 91% N_2_ in RPMI-1640 medium supplemented with 10% human serum in the presence of inhibitors for 48 h without media change. Inhibitors were added from 1000 (DMSO) stocks. After 48 h, the culture medium was removed and replaced with 1% formaldehyde in PBS pH 7.4 for an additional 48 h at room temperature to fix cells. Fixed parasites were transferred into 0.1% Triton-X-100 in PBS containing 1 nM YOYO-1 dye (Molecular Probes). Parasitemia was determined from dot plots (forward scatter vs. fluorescence) ac-quired on a FACS sort flow cytometer using Cell Quest software (Beckton Dickinson). The MIC of compounds was the minimum concentration at which more than 99% of the parasites, relative to the control, were inhibited from developing to schizonts (parasites with six or more chromatin dots)^29^.

### 2.6. *In vivo* antimalaria test

Experimental Design and Treatment of Mice. Methods of Peter et al.^30^ and Kalra et al.^31^ for antiplasmodial assay against *Plasmodium berghei* infection in mice with some modifications were employed. About eighty infected mice were randomly divided into five groups, each having fourteen mice. The inoculum was prepared from a donor mouse with rising parasitemia of 60.42%. After 7 days of infection, animals begin to receive treatment (100 and 200 mg/kg b wt.) of the synthesized compounds (**7a–7n**) for 12 days with constant check of the percentage of parasitemia after a 4-day interval. Artemisinin (100 and 200 mg/kg body weight.) was given to the other mice in group three as positive control, group four was not treated and group five was not infected. All the compounds and the drugs were given orally by using a standard intragastric tube. For all parasitemia determination, blood samples were collected from tail snip of each mouse and thin smears prepared and stained with 10% Giemsa solution. The uniform fields of each stained slide (for each mouse) were examined under microscope with an oil immersion objective of 100X magnification power and average percent of parasitemia was determined.

### 2.7. Haematological analysis

Twenty-four hours before the injecting the compounds, the blood sample of five mice was taken and it was also taken after the last day of treatment, the animals were sacrificed by cervical dislocation and the blood samples were collected by heart puncture. The blood samples for haematological parameters (red blood cell (RBC) count, white blood cell (WBC) count, packed cell volume (PCV), and hemoglobin (HGB)) were collected into EDTA bottles and analyzed using an automated machine (Automated CBC Analyzer: Sysmex KX-21).

### 2.8. Liver function tests (LFTs)

The liver function tests carried out with the blood of the rats fed with the sulfonamide derivatives were aspartate aminotransperase (AST), alanine transaminase (ALT) and alkaline phosphatase (ALP). Standard laboratory procedure according to Reitman and Franke^32^ was used for the determination of parameters.

### 2.9. Renal or kidney function test

Kidney function tests carried out with the blood of the rats fed with the sulfonamide derivatives were, creatinine and albunim. The method reported by Kaplan and Tengv^33^ was used in the determination of creatinine.

### 2.10. *In silico* studies

#### 2.10.1. Physicochemical evaluation

The physicochemical properties used for the evaluation of the drug-likeness of the synthesized compounds were calculated. The molecular descriptors calculated include: molecular weight (MW), partition coefficient (log P), hydrogen bond acceptor (HBA), hydrogen bond donor (HBD), topological polar surface area (TPSA) and number of rotatable bond (NoRB), number of acid (nAc), molar refractivity (MR) and number of atoms (nA). Four receptors were used for this study: two each for antimalarial and antibacterial studies, respectively. The receptors for antimalarial study include Plasmepsin I (PDB ID: 3QS1) and Plasmepsin IV (PDB ID: 1SME) all from *P. falciparum*. The receptors for antibacterial study include penicillin-binding protein 1B (PDB ID: 2Y2G) and *E. coli* DNA gyrase (PDB ID: 5MMN). The co-crystallized inhibitors for each receptor are as follows: 3QS1 – KNI-10006; 1SME – pepstatin A; 2Y2G – alkyl boronate, and 5MMN – 1-ethyl-3-[8-methyly-5–(2-methylpyridin-4-yl)-isoquinolin-3-yl]-urea. The 3D crystal structures of these receptors with their co-crystallized ligands were from the online protein data bank repository (https://www.rcsb.org/). The chemical structures of the synthesized compounds were drawn using ChemSketch. Further preparations of the protein and the ligands were done using the discovery studio. These preparations included deleting of multiple chains, water of crystallization from the protein and energy minimization of the structures. The prepared ligands were docked into the binding cavity of the receptors and their interactions visualized Discovery Studio Visualizer, v16.1.0.15350.

## 3. Results and discussion

**Scheme 1.**
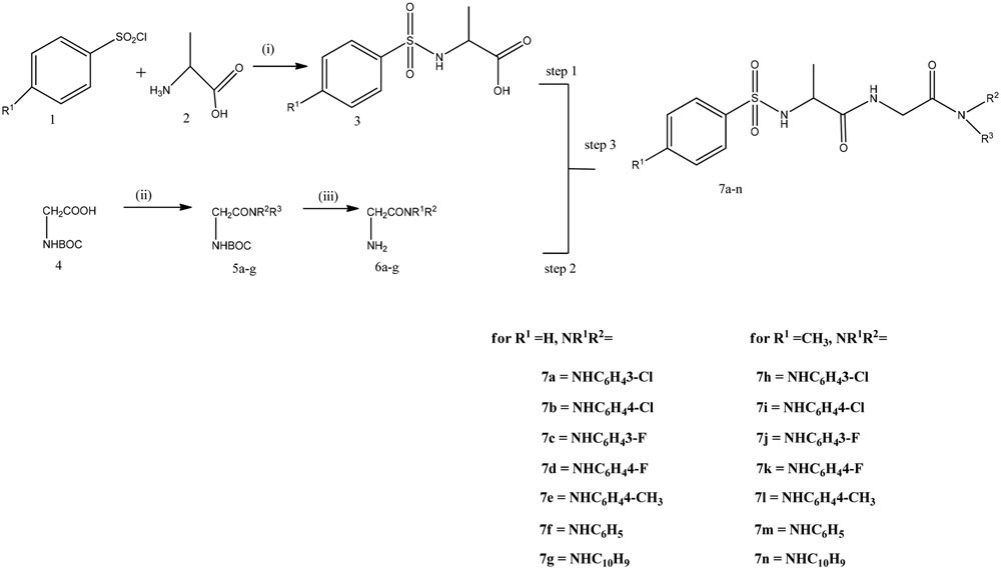
Synthesis of glycinediamide derivatives, reagents and conditions. (i) Na_2_CO_3_, DCM/H_2_O, HCl, 0 °C, r.t, Ph 2, 4 h. (ii) EDCI, HOBt, TEA, DCM, amine,16 h. (iii) 10% TFA in DCM. (V)EDCI, HOBt, TEA or DMAP, 16 h.

The first stage for the preparation of the desired diamide derivative of the glycine is the synthesis of phenylsulphonamido acids by reacting substituted benzenesulphonyl chloride and an amino acid (alanine) in the presence of Na_2_CO_3_ as a base, the acid formed after 4 h reaction and on acidification of the reacting mixture. The diamide derivatives of glycine were synthesized by reacting commercially available boc-protected glycine with amines in the presence of 1-ethyl-3–(3-dimethylaminopropyl) carbodiimide hydrochloride (EDCI), 1-hydroybenzotriazole (HOBT), and triethylamine (TEA) to yield various C-substituted amide derivatives of glycine. (EDCI) alone could not sufficiently activate the carboxylic acid group of glycine and thus addition of (HOBt) was necessary for the coupling reaction. Compound **5** was converted into its salt by addition of 10% trifluoroacetic acid(TFA) in DCM. The salt on further reaction with acid in the presence of peptide coupling agents EDCI, HOBt, TEA or DMAP, gave the desired diamide derivative of the glycine (Scheme 1). The structure of compound **7a–7n** was confirmed by characterization using (FTIR), (^1^HNMR), (^13^NMR). The IR showed strong band at 3361 and 3324 showing the presence of two NH (amide) bond of the derivatives, it also shows strong bonds at 1673 and 1647 for amide carbonyls and a band at 1355 cm^−1^ for sulfonamide group which indicate successful formation of the compounds. ^1^H NMR of **7a** shows the methylene group of glycine exhibiting multiplet at ^δδ^3.5–4.00 due to the interaction CH_2_ and NH of the glycine. There is a doublet at 1.0–1.2 due to the CH AND CH_3_ interaction of the alanine amide. The compound also exhibited multiplet from 7.1–7.9 representing aromatic protons. ^13^C NMR shows strong peak at 168.16 and 172.25 showing the presence of two carbonyl carbons. The appearance of peak at 19.02, 42.99, 53.30 shows the presence of three aliphatic carbon. The peak at 117.82, 118.88, 123.48, 127.00, 129.49, 131.02, 132.89, 133.56, 140.67 and 141.30 shows aromatic carbon.

Over a century ago, Medicinal Chemists have used the physicochemical properties of compounds to predict or estimate pharmacokinetic properties^34^. Drug-likeness has also been used as a parameter to predict the balance among the molecular properties of a compound that influences its pharmacodynamics and pharmacokinetic properties^35^. The absorption, distribution, metabolism, and excretion of drug can be optimized using the physicochemical parameters. The drug-likeness of our compounds were evaluated using Lipinski's rule of five (ro5) and Reckitt's rule. For bioavailability of orally administered drug, ro5 implies that the drug must have molecular weight value of ≤500, hydrogen bond donor ≤5, hydrogen bond acceptor ≤10, and partition coefficient (Log *P*) value ≤5.” Reckitt, which is a modified Lipinski’s rule of 5 (ro5), stated that a likely drug molecule should have an octanol-water partition coefficient (log P) between –0.4 and 5.6, molar refractivity (AMR) between 40 and 130, number of atoms (nA) between 20 and 70, hydrogen bond donor (HBD) ≤ 5 taken as equivalent to the number of –OH and –NH groups, hydrogen bond acceptor (HBA) ≤10 taken as equivalent to the number of oxygen and nitrogen atoms and molecular weight (MW) not more than 500. A violation of more than one of these physicochemical parameters disqualifies a compound from being a likely drug candidate. From Table 1, there was no violation of Lipinski's ro5 and not more than one violation of Reckitt rule. This, therefore all the compounds reported have good balance between compound solubility and its penetration of the lipid bilayers, and hence are likely going to have good oral bioavailability. However, compounds that will serve as substrate for biological transporters do not obey this rule. They can have violations up to 4^36^. This then imply that violations of more than one rule do not totally rule out a compound as a likely drug candidate. Log P shows the degree of lipophilicity of drug molecules, which affects the solubility, absorption, distribution, metabolism, and excretion properties. The log *P* of compounds in Table 1 ranges from 1.37–2.93 which is within the optimal limit. When a drug molecule is highly lipophilic, it will partition into the lipid interior of membranes and is retained there. When log P is higher than the upper limit, the drug molecule will have low solubility whereas in lower log P, the drug has difficulty to penetrate the lipid membranes^37^. It has been reported that the number of rotatable bond (NoRB) influences bioavailability in rats and a cut off of NoRB ≤10 is recommended for good oral bioavailability^38^. All the compounds reported here have NoRB ≤10. Total polar surface area (TPSA) has also been used as a surrogate property for cell permeability. A molecule with TPSA ≤140 Å^2^ would be able to permeate the cell. Again, all the compounds had TPSA less than 140 and as such can permeate the cell membranes. To examine whether or not a drug molecule will be able to permeate through the blood brain barrier (BBB) for the treatment of central nervous system (CNS) related diseases, certain parameters in the BBB likeness are considered. BBB likeness states that a CNS drug must have HBA value in the range of 8–10, the MW must be in the range of 400–500 and the number of acid must be zero. All the synthesized compounds reported herein fulfilled the conditions and as such can cross the blood brain barrier and might be useful as to treat brain inflammations.

**Table 1.** Physicochemical properties of the synthesized compounds.

Mol	HBA	aAc	aA	HBD	Nrb	logP (o/w)	MR	TPSA	MW	LNV	RNV
**7a**	4	0	44	3	9	2.00	10.17	104.37	395.87	0	1
**7b**	4	0	44	3	9	1.96	10.17	104.37	395.87	0	1
**7c**	4	0	44	3	9	1.56	9.74	104.37	379.41	0	1
**7d**	4	0	44	3	9	1.52	9.74	104.37	379.41	0	1
**7e**	4	0	47	3	9	1.67	10.12	104.37	375.45	0	1
**7f**	4	0	44	3	9	1.37	9.67	104.37	361.42	0	1
**7g**	4	0	50	3	9	2.63	11.29	104.37	411.48	0	1
**7h**	4	0	47	3	9	2.30	10.63	104.37	409.89	0	1
**7i**	4	0	47	3	9	2.26	10.63	104.37	409.89	0	1
**7j**	4	0	47	3	9	1.86	10.20	104.37	393.44	0	1
**7k**	4	0	47	3	9	1.82	10.20	104.37	393.44	0	1
**7l**	4	0	50	3	9	1.97	10.57	104.37	389.48	0	1
**7m**	4	0	47	3	9	1.67	10.12	104.37	375.45	0	1
**7n**	4	0	53	3	9	2.93	11.74	104.37	425.51	0	1

MW: molecular weight; HBA: hydrogen bond acceptor; HBD: hydrogen bond donor; TPSA: total polar surface area; nRB: number of rotatable bond; nAc: number of acid; MR: molar refractivity; nA: number of atoms; LNV: Lipinski’s number of violations; RNV: Reckitt’s number of violation.

To gain further insight in the binding interactions of our compounds and the drug receptors, we carried out molecular docking studies. Table 2 shows the free binding energy, Δ*G* (kcal/mol) of the compounds against each selected drug receptor. These Δ*G* were compared to both the co-crystallized inhibitor and the standard drug. All the compounds studied had a good binding affinity with all the four drug receptors used in this study. Compound **7g** had a better binding affinity (−12.91 kcal/mol) with plasmepsin I receptor from *P*. *falciparum* (3QS1) when compared to the standard drug, chloroquine (−11.67 kcal/mol). Its binding affinity was not significantly different from the co-crystallized inhibitor. Similarly compounds **7a** and **7h** had a better binding affinity with plasmepsin II receptor from *P*. *falciparum* (1SME) when compared to both the co-crystallized inhibitor and the standard drug. Compounds **7b** and **7n** showed good binding affinities with penicillin-binding protein 1B (2Y2G) and E. coli DNA gyrase (5MMN).

**Table 2.** Free binding energy (ΔG, kcal/mol) of compounds.

		Malaria Receptors		Bacterial receptors	
S/N	Compound	3QS1	1SME	2Y2G	5MMN
1	**7a**	−11.96	−12.61	−12.88	−10.52
2	**7b**	−11.66	−11.37	−13.43	−10.58
3	**7c**	−12.09	−11.75	−13.09	−10.38
4	**7d**	−11.83	−11.78	−13.34	−10.98
5	**7e**	−12.16	−12.51	−12.48	−10.72
6	**7f**	−12.50	−11.43	−12.98	−10.62
7	**7g**	−12.91	−12.55	−13.69	−10.75
8	**7h**	−12.34	−12.61	−12.59	−9.95
9	**7i**	−11.60	−11.24	−12.09	−9.90
10	**7j**	−11.65	−11.89	−12.41	−10.98
11	**7k**	−12.01	−11.72	−12.24	−10.81
12	**7l**	−11.63	−11.99	−11.91	−10.62
13	**7m**	−12.22	−12.24	−12.60	−10.83
14	**7n**	−11.99	−11.81	−13.30	−11.11
15	Native ligand	−13.45	−8.82	−13.24	−11.50
16	Standard drug	−11.67	−11.29	−12.32	−9.92

Standard drugs: Chloroquine for antimalarial and penicillin for antibacterial.

**Figure 1.**
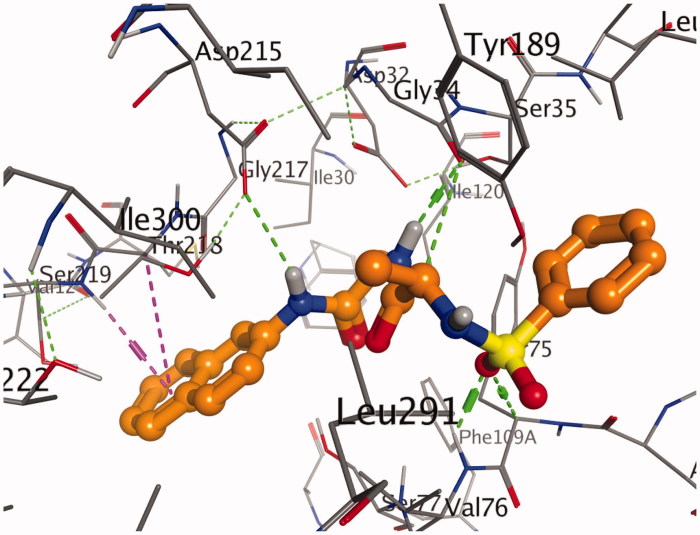
A stereoview showing the active site of the **7g**-2Y2G complex. **7g** is shown in ball-and-stick model (orange). The amino acids are shown as lines (black). Hydrogen and π-H bonds between **7g** and the protein are shown as green and purple dotted lines, respectively.

**Figure 2.**
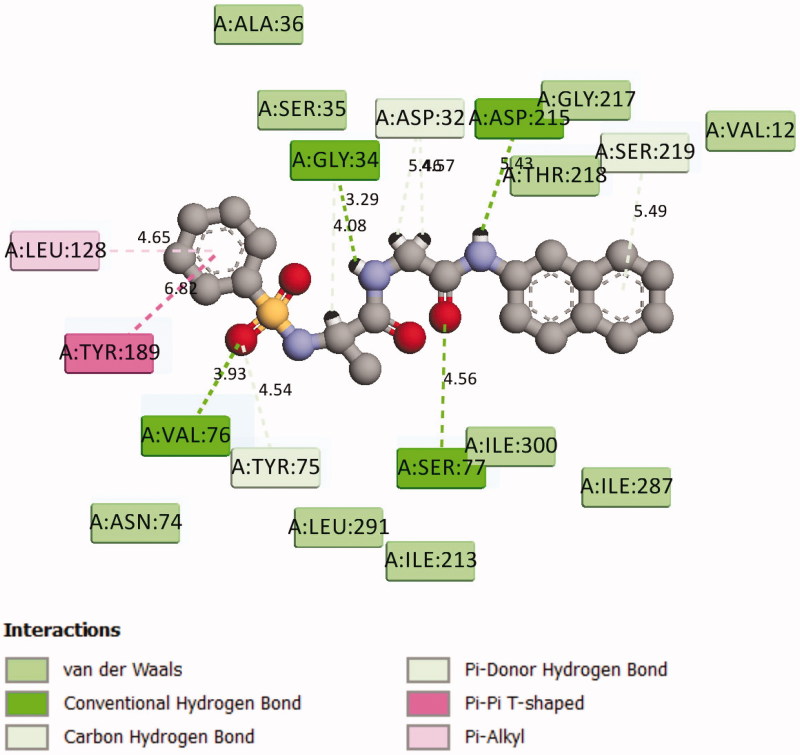
Representation of the chemical interactions of **7g** with the amino acid residues of 3QS1.

**Figure 3.**
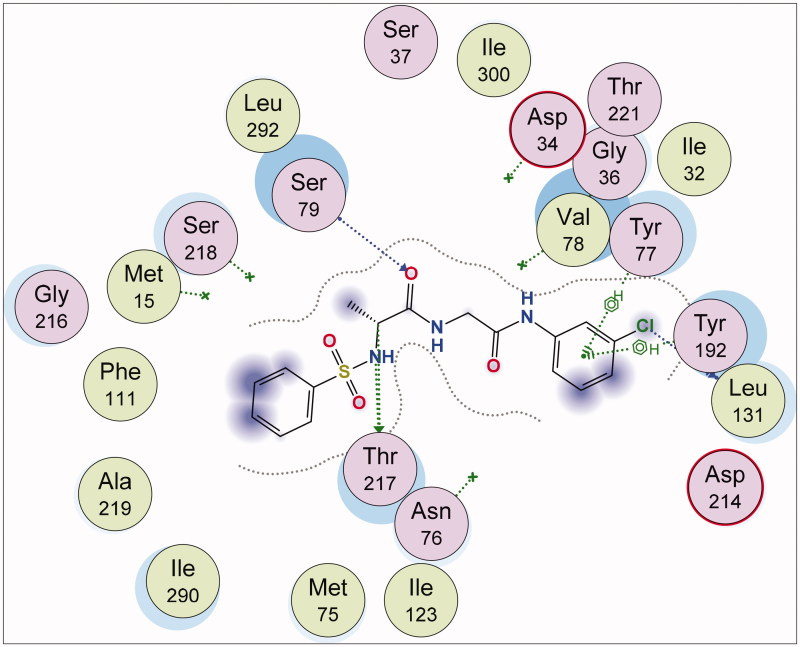
2D Representation of the chemical interactions of **7a** with the amino acid residues of 1SME.

**Figure 4.**
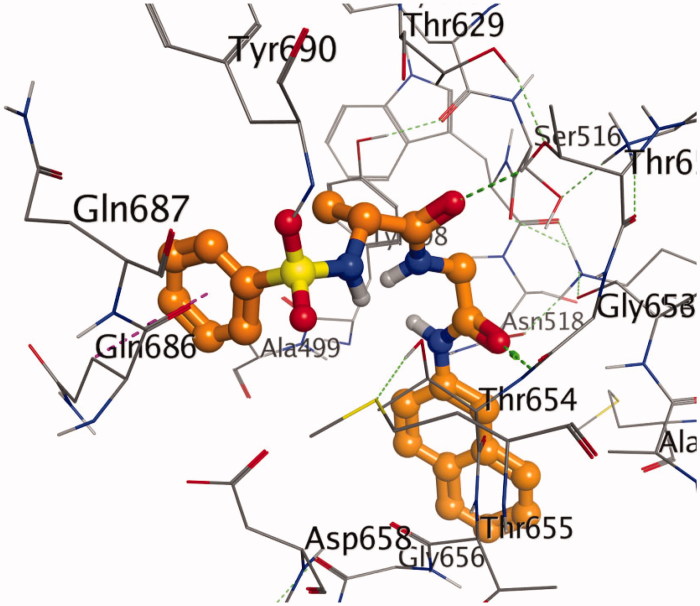
A stereoview showing the active site of the **7g**-2Y2G complex. **7g** is shown in ball-and-stick model (orange). The amino acids are shown as lines (black). Hydrogen and π-H bonds between **7g** and the protein are shown as green and purple dotted lines, respectively.

**Figure 5.**
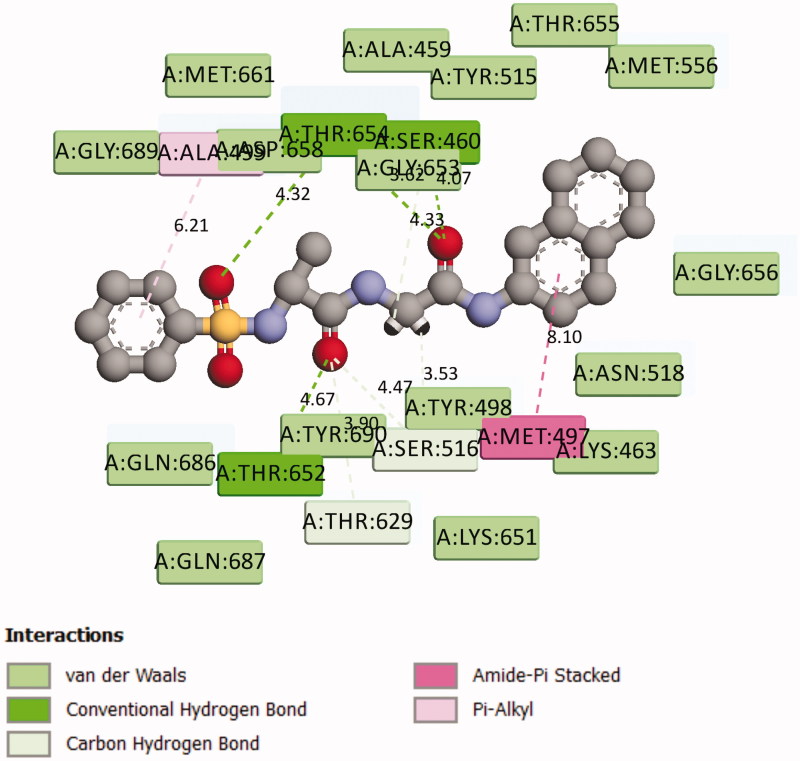
Representation of the chemical interactions of **7g** with the amino acid residues of 2Y2G.

**Figure 6.**
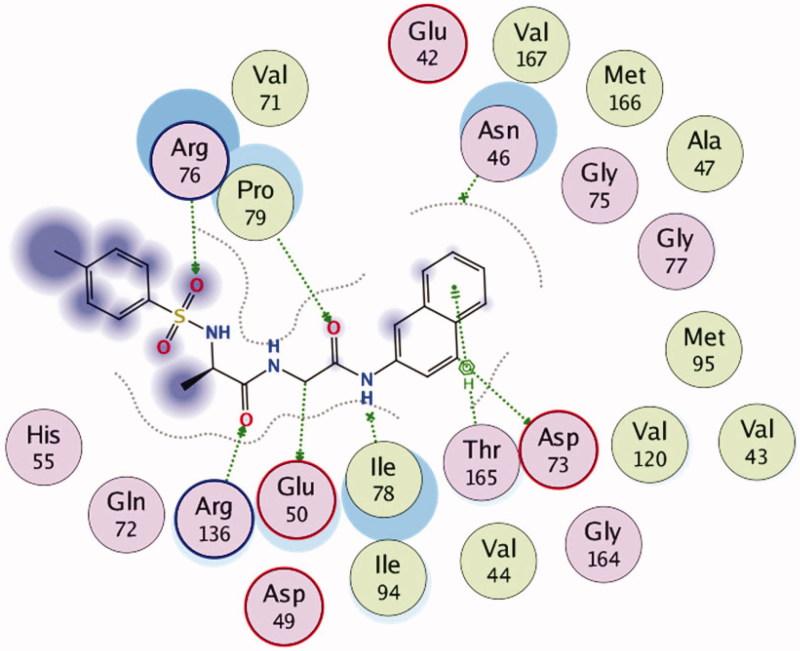
2D Representation of the chemical interactions of **7n** with the amino acid residues of 5MMN.

These findings have further necessitated in-depth studies of **7g**-3QS1, **7a**-1SME, **7g**-2Y2G and **7n**-5MMN complexes with a view of understanding their binding interactions with the receptors. Figures 1–6 show the various molecular interactions of these compounds woth drug receptors.

Compound **7g** makes multiple contacts of different natures with the active site residues of 3QS1 as shown in Figures 1 and 2. While Figure 1 specifically showed the binding pose of **7g** in the binding cavity of 3QS1, Figure 2 illustrated the details of the chemical interactions involved. There were four different H-bond interactions observed. The O-atom of **7g** and interacted with SER 77 at an intermolecular distance of 4.56 Å. Another O-atom interacted with VAL 76 (3.96 Å). N-13 interacted with O of GLY 34 (3.29 Å) while N-16 interacted with OD2 of ASP 215 (5.43 Å). There was π-π T shaped interaction between the aromatic 6-membered ring of **7g** and TYR 189. π-alkyl interaction occurred between the same 6-membered ring and LEU 128 at an intermolecular distance of 4.65. There were also four different π-donor H bonding involving TYR 75, 2(ASP 32) and SER 219. There were other conserved amino acid residues which were involved in hydrophobic interactions with **7g** as shown in Figure 2. These numerous and significant interactions of compound **7g** explain the observed high binding affinity with 3QS1.

In Figure 3, different atoms of **7a** interacted with the 1SME receptor. N-10 and C-11 of **7a** interacted, respectively, with the OG1 THR 217 through H-donor interactions. The distance and energy of interaction of N-10 and THR 217 are 2.94 Å and −0.6 kcal/mol, respectively, while those of C-11 and THR 217 are 3.07 Å and −0.2 kcal/mol, respectively. Also, Cl-23 of **7a**, through H-donor interaction, had a contact with O LEU 131. There were two different H-acceptor interactions: 1. Between O-25 atom of **7a** and N SER 79 at an intermolecular distance of 3.20 Å; 2. Between the same O-25 atom and OG SER 79 at a distance and energy of 2.96 Å and −2.0 kcal/mol, respectively. The chlorobenzene ring of compound **7a** was involved with two different π-H bonding interactions with the amino acid residues of 1SME. Firstly, it interacted with CA TYR 77 at an intermolecular distance of 4.69 Å, and secondly, it interacted with the OH TYR 192 at 3.42 Å.

Figure 4 shows the binding pose of compound **7g** in the binding cavity of 2Y2G while Figure 5 represents the chemical interactions involved in the 2Y2G-**7g** complex. The various chemical interactions contribute to the observed strong binding affinity between compound **7g** and the penicillin-binding receptor. Four different H-bonding interactions were observed. Firstly, the O-27 atom of the compound combined with THR 654 through an intermolecular distance of 4.32 Å. Secondly, O-28 atom combined with THR 652 (4.67 Å). Thirdly, another O-atom interacted with THR 654 (4.33 Å), and lastly, the same O-atom interacted with SER 460 (4.07 Å). There was also found amide-π stacked interaction. MET 497 interacted with the π-electrons of the naphthalene ring. Other forms of interactions between compound **7g** and 2Y2G are shown in Figures 4 and 5.

Figure 6 shows the 2D representation of the chemical interactions of **7n** with the amino acid residues of 5MMN. A lot of significant interactions were involved. There were two different H-donor interactions observed. C-14 atom of **7n** interacted with OE1 GLU 50 (distance of interaction = 3.50 Å). Also, C-19 of the compound interacted with OD2 ASP 73 (distance of interaction = 3.37 Å). O-9 atom of **7n** interacted through H-acceptor interaction with NH1 ARG 76. Three other H-acceptor interactions occurred between O-9, O-27 and O-28, and NH2 ARG 76, CD PRO 79 and NH1 ARG 136, respectively. Finally, there was an H-bonding interaction between the 6-membered aromatic ring of **7n** and the CG2 THR 165 (4.49 Å).

### 3.1. Antimicrobial activities

The antibacterial activities of the test compounds are shown in Table 3. The compound showed great antibacterial activities against all the micro-organism. Some of the test compound showed better activities against the bacteria than the control drug(ciprofloxacin). Compound **7b**, **7f**, **7g** and **7m** showed greater activity against Bacillus than ciprofloxacin. In all the bacteria used, some of the test compound have better activity than ciprofloxacin. In fungi, the test compounds showed great activities against *Aspergilus* as well as against *Candida*. Some of the compounds were more active than fluconazole(standard drug used) as shown in Table 3.

**Table 3.** MIC (minimum inhibitory concentration) mg/ml.

Comps	*B. subtilis*	*E. coli*	*S. typhi*	*P. aeruginosa*	*K. pneumoniae*	*S. aureus*	*C. albicans*	*A. niger*
**7a**	0.219	0.489	0.199	0.398	0.251	4.677	0.0561	1.585
**7b**	0.129	0.467	0.602	0.257	1.69	0.288	0.135	0.1000
**7c**	3.467	0.372	2.691	0.457	0.129	–	3.467	–
**7d**	0.194	0.521	0.476	0.295	0.177	0.489	0.0343	1.995
**7e**	–	6.327	0.194	3.802	–	0.389	0.0288	2.089
**7f**	0.155	0.131	4.677	0.354	5.011	0.190	0.214	0.1047
**7g**	0.141	–	0.079	–	0.166	–	0.0297	0.158
**7h**	0.214	0.239	1.288	0.616	5.248	0.234	0.034	0.0316
**7i**	0.214	0.239	1.288	0.616	5.248	0.234	0.034	0.0316
**7j**	0.245	0.275	0.129	0.380	0.083	–	0.371	–
**7k**	0.371	0.316	3.161	3.162	0.079	0.387	0.0631	0.316
**7l**	0.245	0.169	0.057	0.169	0.107	0.115	0.174	0.123
**7m**	0.129	0.123	0.057	0.144	0.138	0.229	7.94	0.056
**7n**	–	0.123	0.617	0.129	–	–	0.155	0.039
CIP	0.184	0.131	0.128	0.232	0.113	0.252	–	–
FLU	–	–	–	–	–	–	0.0217	0.0471

– : No activity; CIP means Ciprofloxacin; FLU means Fluconazole.

### 3.2. *In vitro* antimalarial

To determine the *in vitro* antimalarial activity of the primary sulfonamides, minimum inhibition assay was employed using a chloroquine sensitive *P. falciparum* malaria parasite line (Table 4) Chloroquine and artemesinin were used as standard antimalarial drugs to compare those of the test compounds. Compounds **7d**, **7e** and **7h** (MIC 0.03, 0.03 and 0.05 mM) displayed activity better than chloroquine (MIC 0.08 mM), whereas about five of the total newly synthesized sulfonamides inhibited the activity of *P. falciparum* parasite at lower MIC range than artemisinin On the overall, compound **7h** emerged as having the best activity among the newly synthesized compound and relative to both considered standard antimalarial drugs.

**Table 4.** *In vitro* antimalarial study.

Compounds	MIC (µM)
**7a**	0.37
**7b**	0.87
**7c**	0.72
**7d**	0.08
**7e**	0.05
**7f**	2.32
**7g**	3.23
**7h**	0.03
**7i**	0.77
**7j**	0.88
**7k**	0.53
**7l**	0.93
**7m**	1.87
**7n**	2.34
Chloroquine	0.08
Artemisimin	0.72

### 3.3. *In vivo* antimalarial

To determine the in vivo activity, the compounds were tested against *P. berghei* NK(65 Strain) infection mice., the animals were obtained from Nigeria institute for Trypanosoma and onchocercarioses Research (NTIR) Vom, Plateau State Nigeria. The permission and approval for the use of animals in this experiment were granted by the Animal Ethics Committee, Federal College of Vetrenary Medical Laboratory, Vom, Plateau State. Method of Peter et al.^30^, for antiplasmodial assay against *P. berghei* in mice with some modification were employed. Artemisimin was used as treated control of the experiments. The percentage inhibition of parasite multiplication was calculated comparing the treated group with untreated group by means of the following formula^39^ [(A − B)/A] × 100; where A = parasitaemia in the untreated group and B = parasitemia in the test group. Compounds that reduced parasitemia by 40% were considered active, whereas those that reduce parasitaemia by 30–40% or less than 30% were deemed partially active and inactive, respectively^40^. Most of the compounds were active against *P. berghei* after 7 days post-infection by reducing the parasiteamia by at least 40% when in a dose of 100 mg/kg (Table 5). The inhibition of parasitaemia increased on 12th day post infection for almost all the compounds. The control drug showed activities on all the mice by inhibiting the parasitaemia growth more than 40%. However in consideration of the compounds ability to inhibit parasitaemia to the control drug. Compound **7d**, **7e**, **7j**, **7h** and **7e** have almost the same or better activity compared to the artemisinin. These compounds should be considered for further studies because of its promising activities.

**Table 5.** Percentage inhibition of parasite in mice.

Comp	% inhibition 7 days post-treatment (100 mg/kg)	% inhibition 7 days post-treatment (200 mg/kg)	% inhibition 12 days post-treatment (100 mg/kg)	% inhibition 12 days post-treatment (200 mg/kg)
**7a**	36.23	52.11	49.97	61.37
**7b**	51.33	57.62	55.23	53.4
**7c**	48.32	40.22	51.33	48.71
**7d**	53.62	48.73	81.72	55.98
**7e**	62.34	40.22	66.71	50.71
**7f**	38.23	70.97	41.87	73.29
**7g**	27.54	54.69	36.83	51.93
**7h**	61.36	49.85	57.77	55.22
**7i**	51.73	66.57	46.37	81.38
**7j**	77.93	77.36	82.68	73.55
**7k**	40.35	54.92	44.28	56.43
**7l**	38.23	60.72	41.73	74.62
**7m**	33.46	71.36	29.33	73.98
7n	47.8	63.8	49.11	71.66
Arte	75.67	78.87	86.22	82.34
NTC	–	–	–	–
NIC	–	–	–	–

Arte: Artemisinin; NIT: Non treated control; NIC: Non infected control.

### 3.4. Liver function tests evaluation

Liver function tests are a group of blood tests that detect inflammation and damage to the liver. They can check how well the liver is working. The liver function tests studied in this research are AST, ALT and ALP. It is observed from Table 6 that the administration of 10 mg/kg of the sulfonamide to the tested mice did not show significant increment or decrease in the serum levels of AST, ALT and T.P when compared with the control.

**Table 6.** Liver function test.

	ALT (µ/L)		AST (µ/L)		T.P. (g/dl)		Bilirubin (mg/dl)		Glucose (g/l)	
Comp	Before	After	Before	After	Before	After	Before	After	Before	After
**7e**	24.0	22.0	64.0	60.5	5.8	4.5	0.3	0.2	5.8	5.0
**7d**	24.2	22.5	64.0	68.5	5.7	4.0	0.3	0.2	5.8	5.5
**7j**	26.3	25.5	65.0	60.0	6.0	5.8	0.4	0.2	5.9	5.7
**7h**	25.4	24.5	65.0	69.5	6.0	5.5	0.4	0.3	5.9	5.5
**7c**	20.5	23.5	62.0	61.5	5.8	5.5	0.3	0.5	5.6	5.0
Control	24.2		65.5		5.9		0.3		6.3	

### 3.5. Kidney function test evaluation

Kidney function tests are common lab tests used to evaluate how well the kidneys are working. In this research work, the kidney function tests used are albumine, creatinine. In the mice fed with 10 mg/kg of sulfonamide derivatives, there were no significant changes for the serum levels of creatinine and albumin when compared with the control (Table 7).

**Table 7.** Kidney function test.

	Creatinine (mg/dl)		Albumin (g/dl)	
Compound	Before	After	Before	After
**7e**	0.3	0.3	4.1	3.5
**7d**	0.4	0.3	4.0	3.5
**7j**	0.4	0.3	3.9	4.0
**7h**	0.3	0.3	3.9	3.9
**7c**	0.3	0.3	3.5	3.0
Control	0.3		3.9	

#### 3.6. Haematological analysis

The blood samples for haematological parameters (red blood cell (RBC) count, white blood cell (WBC) count, packed cell volume (PCV), and haemoglobin (HGB)) were collected into EDTA bottles and analyzed using an automated machine (Automated CBC Analyzer: Sysmex KX-21) (Table 8). From the analysis, even though there is a decrease in the value of RBC, PCV and HB, it is observed that there is no significant changes in the parameters that were analyzed for the control and the test compounds.

**Table 8.** Heamotological analysis before and after treatment.

	RBC (mm^3^)		PCV (%)		WBC (mm^3^)		HB (g/dl)	
Comp	Before	After	Before	After	Before	After	Before	After
**7e**	6.85 × 10^6^	6.0 × 10^6^	36	35	12.2 × 10^3^	10.5 × 10^3^	14.1	11.0
**7d**	6.82 × 10^6^	6.8 × 10^6^	37	34	13.5 × 10^3^	9.5 × 10^3^	13.5	10.5
**7j**	7.1 × 10^6^	6.5 × 10^6^	39	34	8.0 × 10^3^	9.1 × 10^3^	13.5	10.0
**7h**	7.3 × 10^6^	5.9 × 10^6^	39	34	9.4 × 10^3^	9.3 × 10^3^	13.4	10.0
**7c**	7.5 × 10^6^	6.0 × 10^6^	38	33	10.3 × 10^3^	9.0 × 10^3^	14.5	9.8

## 4. Conclusion

Fourteen new ala-gly dipeptide sulfonamide carboxamides derivatives were synthesis, characterization. The spectral data confirmed the successful preparation of these derivatives. The *in vitro* antibacterial activity of the compounds against some clinically isolated bacteria strains showed varied activities with some of the new compounds showing better activities against the bacteria and the fungi more than the reference drug ciprofloxacin and fluconazole. Compound **7b**, **7f**, **7g** and **7m** showed greater activity against Bacillus than ciprofloxacin. In all the bacteria used, some of the test compound has better activity than ciprofloxacin. In fungi, the test compounds showed great activities against *Aspergilus* as well as against *Candida.* The compounds exhibited antimalarial property *in vitro.* Compounds **7d**, **7e** and **7h** (MIC 0.03, 0.03 and 0.05 mM) displayed activity better than chloroquine (MIC 0.08 mM). **7h** possessed the least MIC of 0.03 µM against *Plasmodium falciparium.* The compounds also exhibited antimalaria activity *in vivo* against *P. berghei* in mice. Most of the compounds were active against *P. berghei* after 7 days post-infection by reducing the parasiteamia by at least 40% when in a dose of 100 mg/kg (Table 5). The inhibition of parasitaemia increased on 12th day post infection for almost all the compounds. The control drug showed activities on all the mice by inhibiting the parasitaemia growth more than 40%. However, in consideration of the compounds ability to inhibit parasitaemia to the control drug, compounds **7d**, **7e**, **7j**, **7h** and **7e** have almost the same or better activity compared to the artemisinin. Compound Molecular docking revealed significant chemical interactions of the compounds with different receptors resulting in high binding affinity. From the haematological analysis, even though there is a decrease in the value of (RBC), (PCV) and (HB), it is observed that there are no significant changes in the parameters that were analyzed for the control and the test compounds. The studies on the liver and kidney showed no significant damage to the organs by the new compounds and it is recommended that more research should be carried out on these compounds as it showed great potential for antimicrobial and antimalarial properties.

## Supplementary Material

Supplemental Material
