# Supplementary material for: New peptide derived antimalaria and antimicrobial agents bearing sulphonamide moiety
Source: J Enzyme Inhib Med Chem. 2019 Aug 8;34(1):1388–99. doi: 10.1080/14756366.2019.1651313 (PMC6713104; doi:10.1080/14756366.2019.1651313)
Supplement: Supplemental Material [file IENZ_A_1651313_SM2427.zip › 7k 13C.docx]

7k
