# Supplementary figures and images for: New peptide derived antimalaria and antimicrobial agents bearing sulphonamide moiety
Source: J Enzyme Inhib Med Chem. 2019 Aug 8;34(1):1388–99. doi: 10.1080/14756366.2019.1651313 (PMC6713104; doi:10.1080/14756366.2019.1651313)

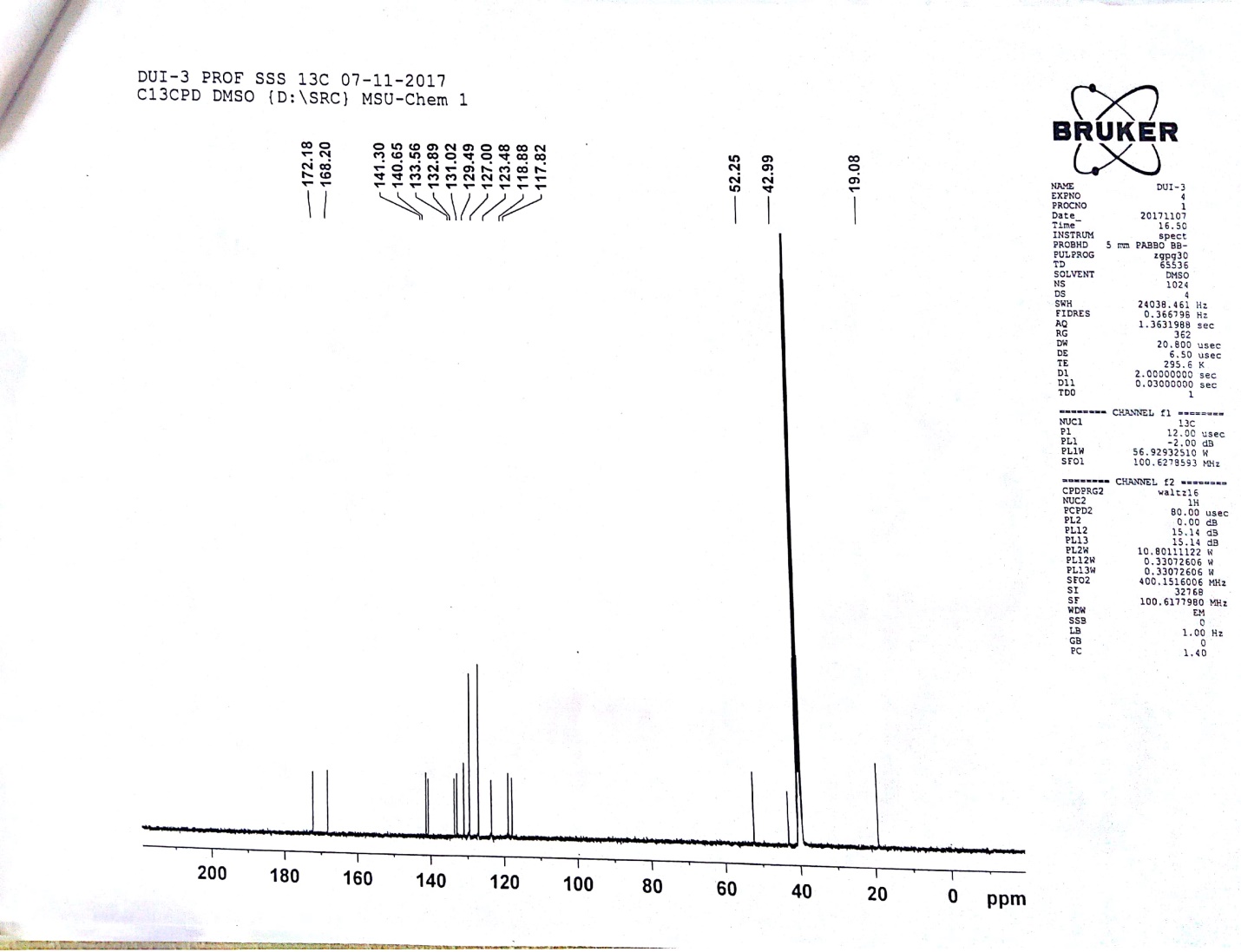


7a

Supplement: Supplemental Material [file IENZ_A_1651313_SM2427.zip › 7a 13C.docx]

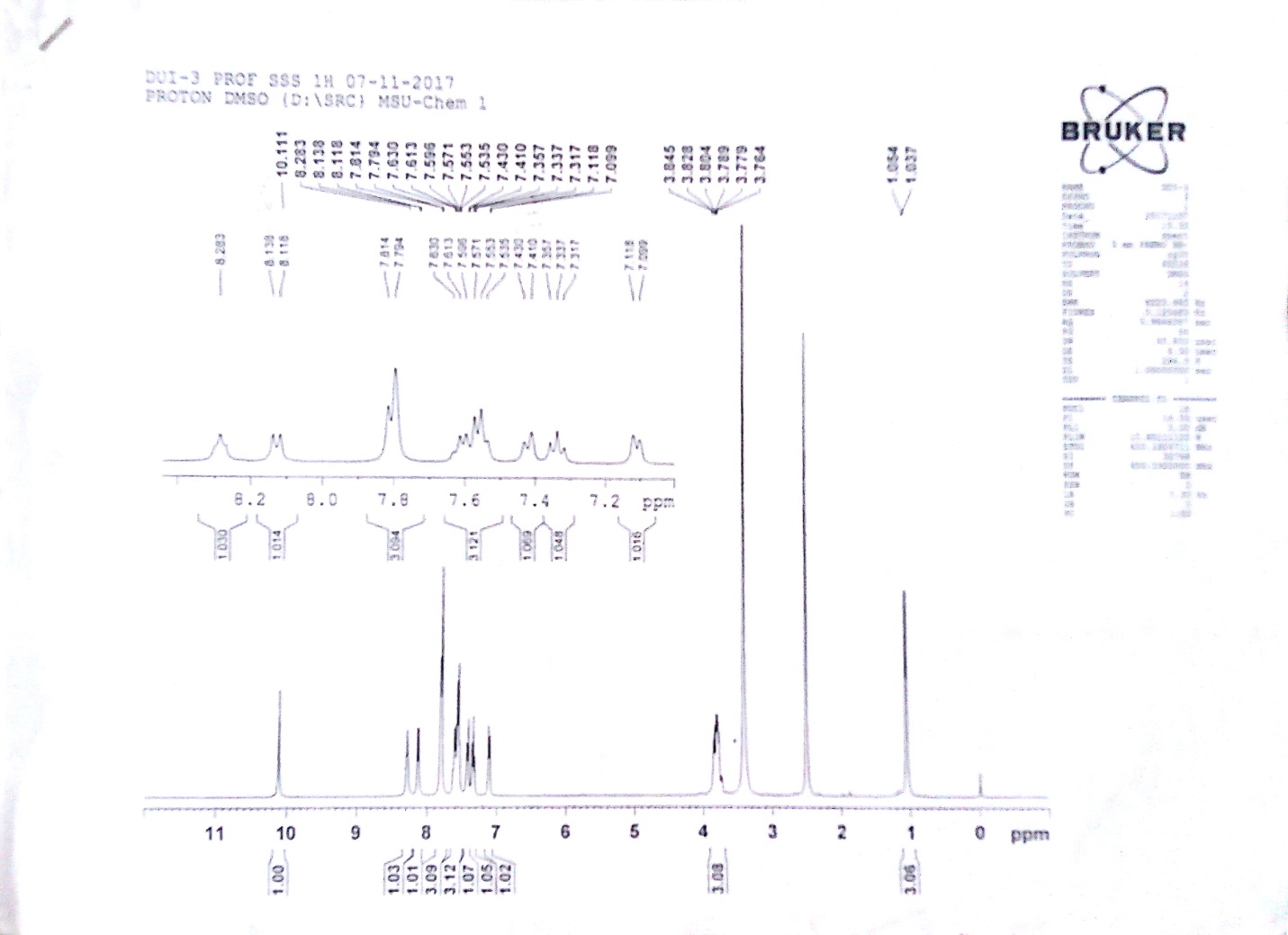


7a

Supplement: Supplemental Material [file IENZ_A_1651313_SM2427.zip › 7a 1H.docx]

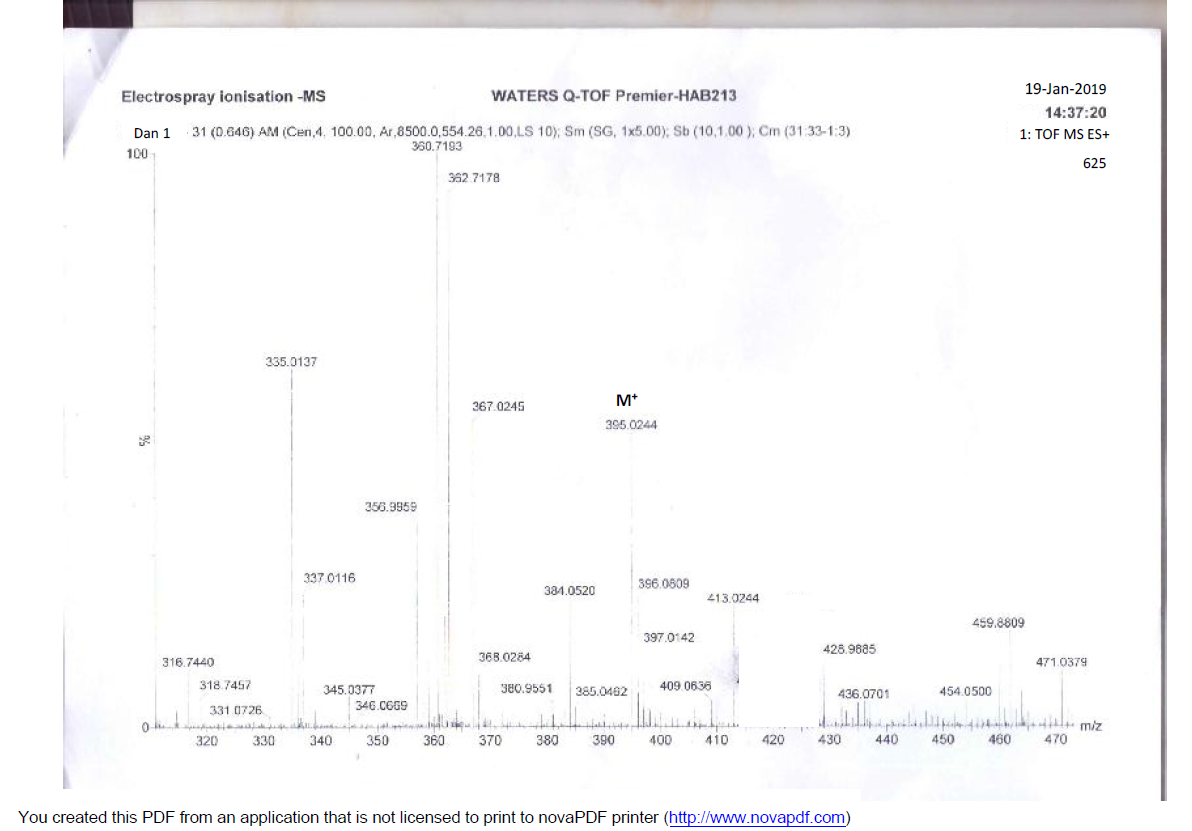


7a

Supplement: Supplemental Material [file IENZ_A_1651313_SM2427.zip › 7a.docx]

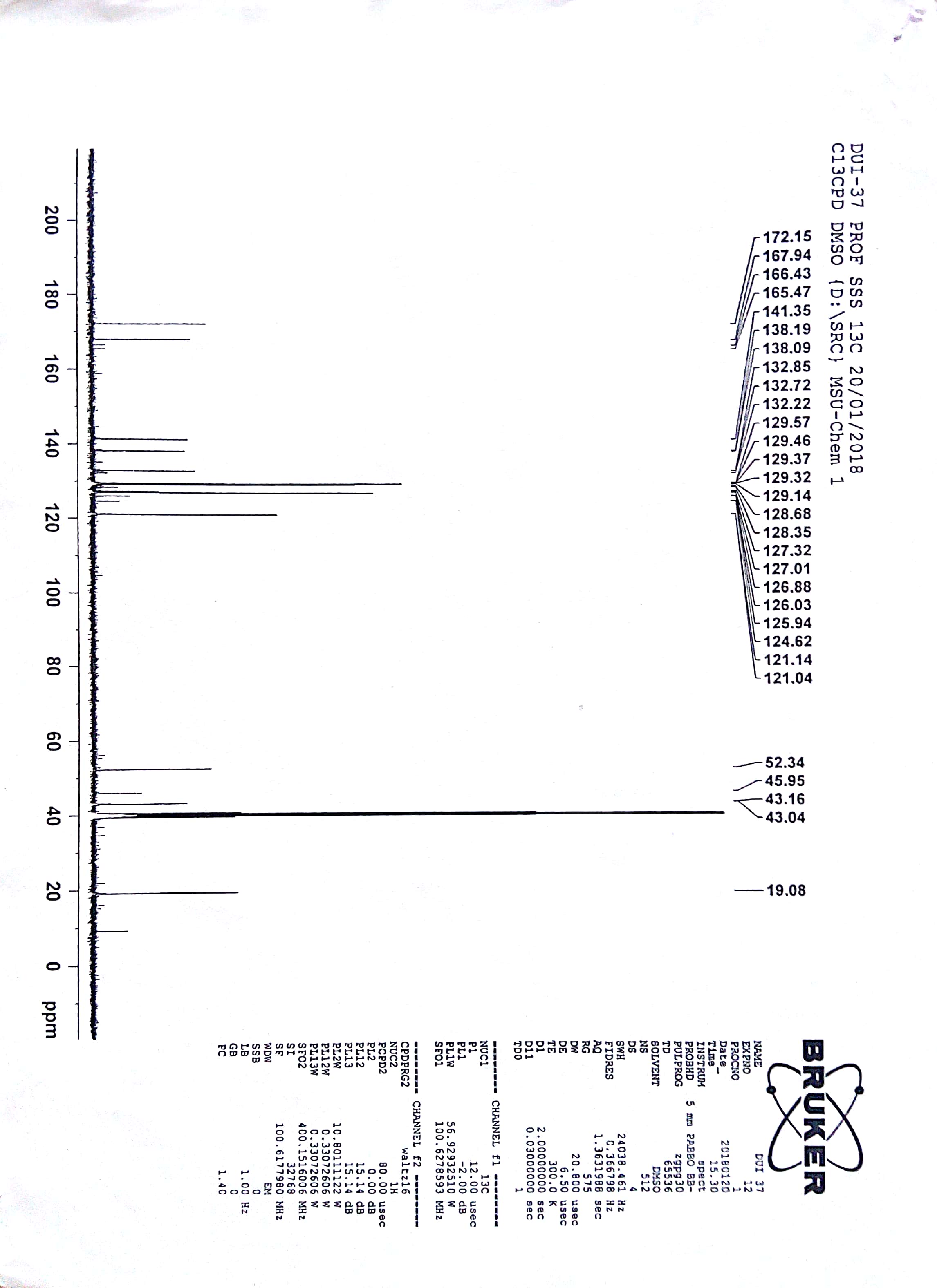


7b

Supplement: Supplemental Material [file IENZ_A_1651313_SM2427.zip › 7b 13C.docx]

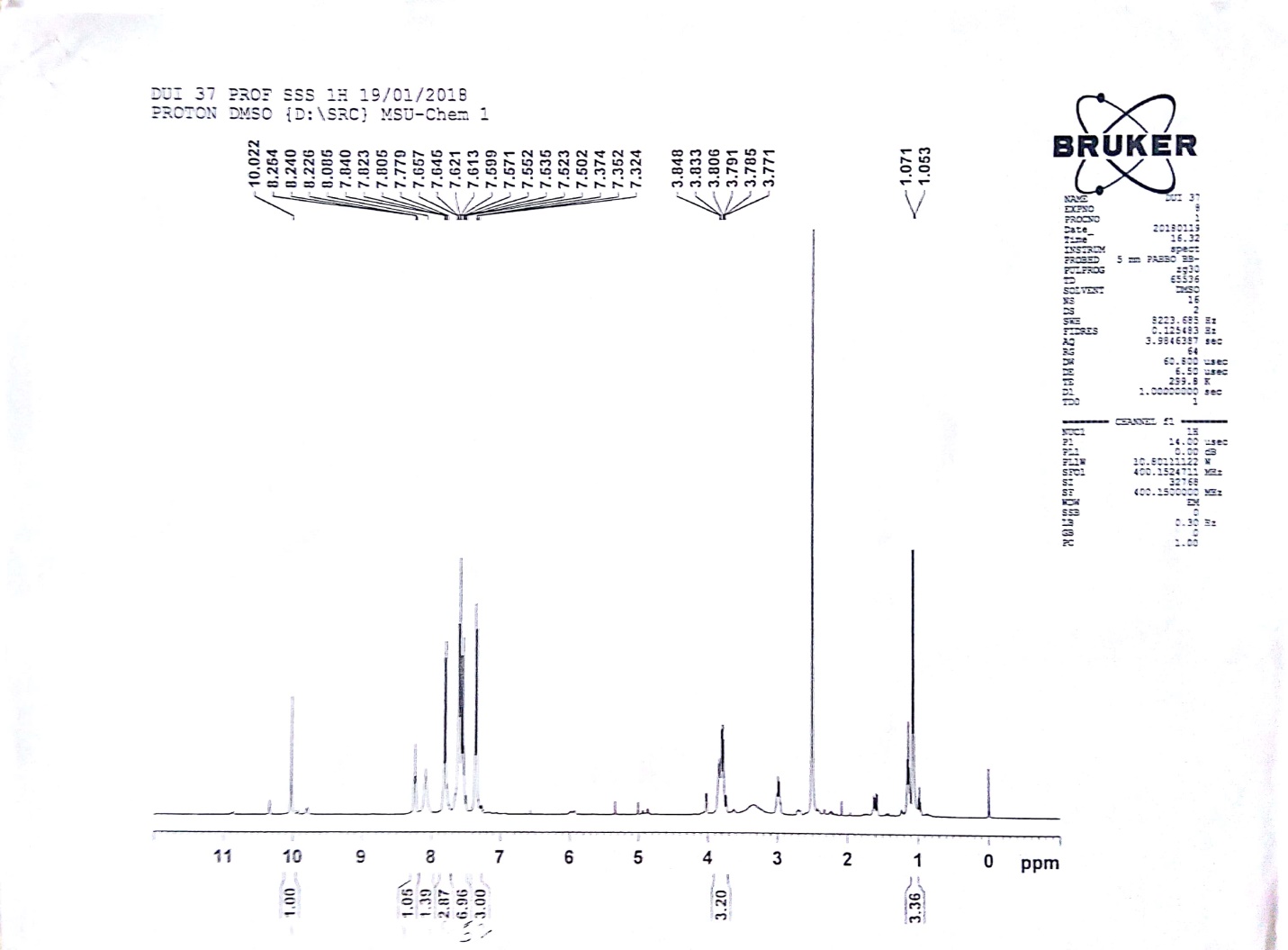


7b

Supplement: Supplemental Material [file IENZ_A_1651313_SM2427.zip › 7b 1H.docx]

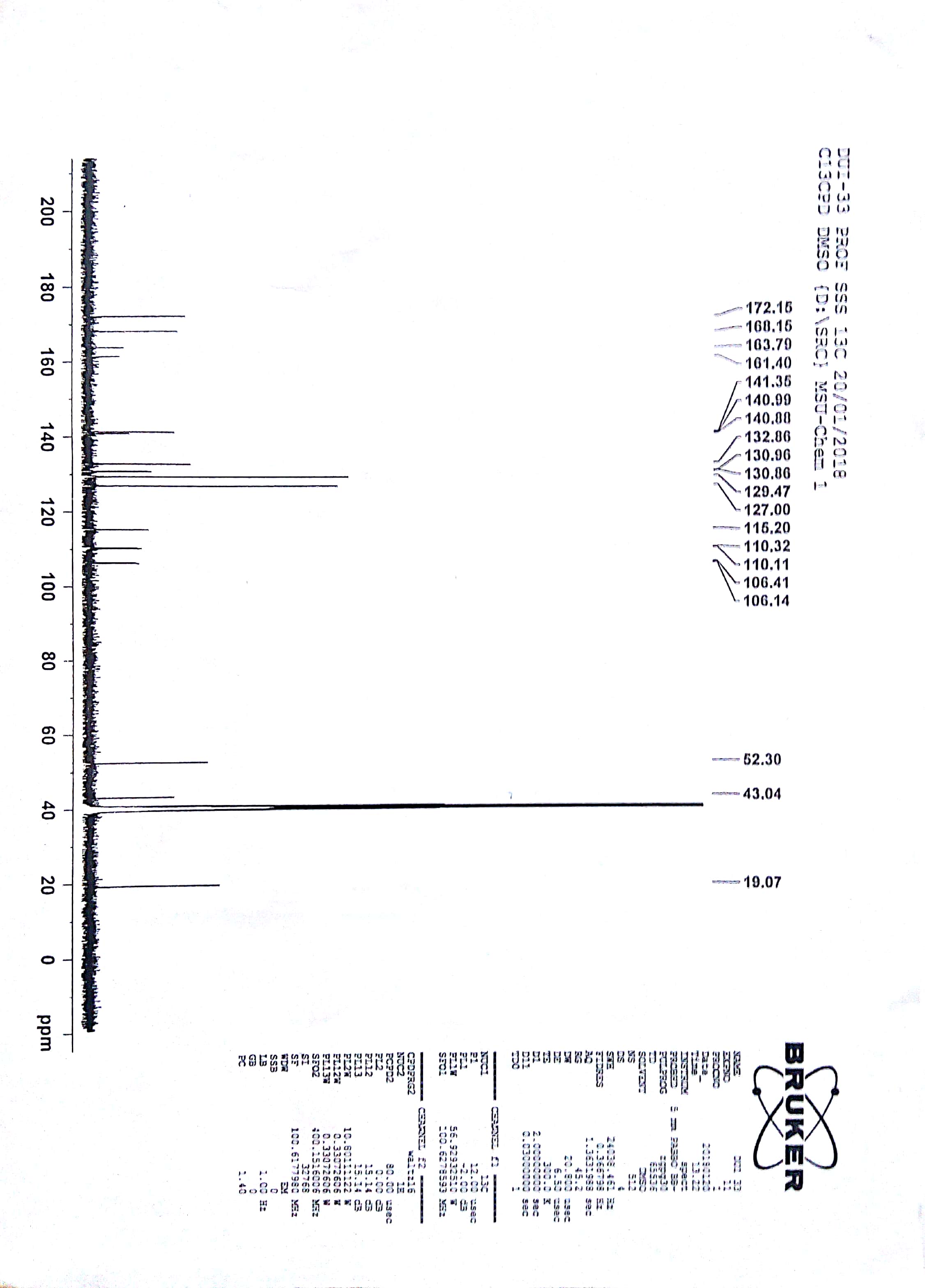


7c

Supplement: Supplemental Material [file IENZ_A_1651313_SM2427.zip › 7c 13C.docx]

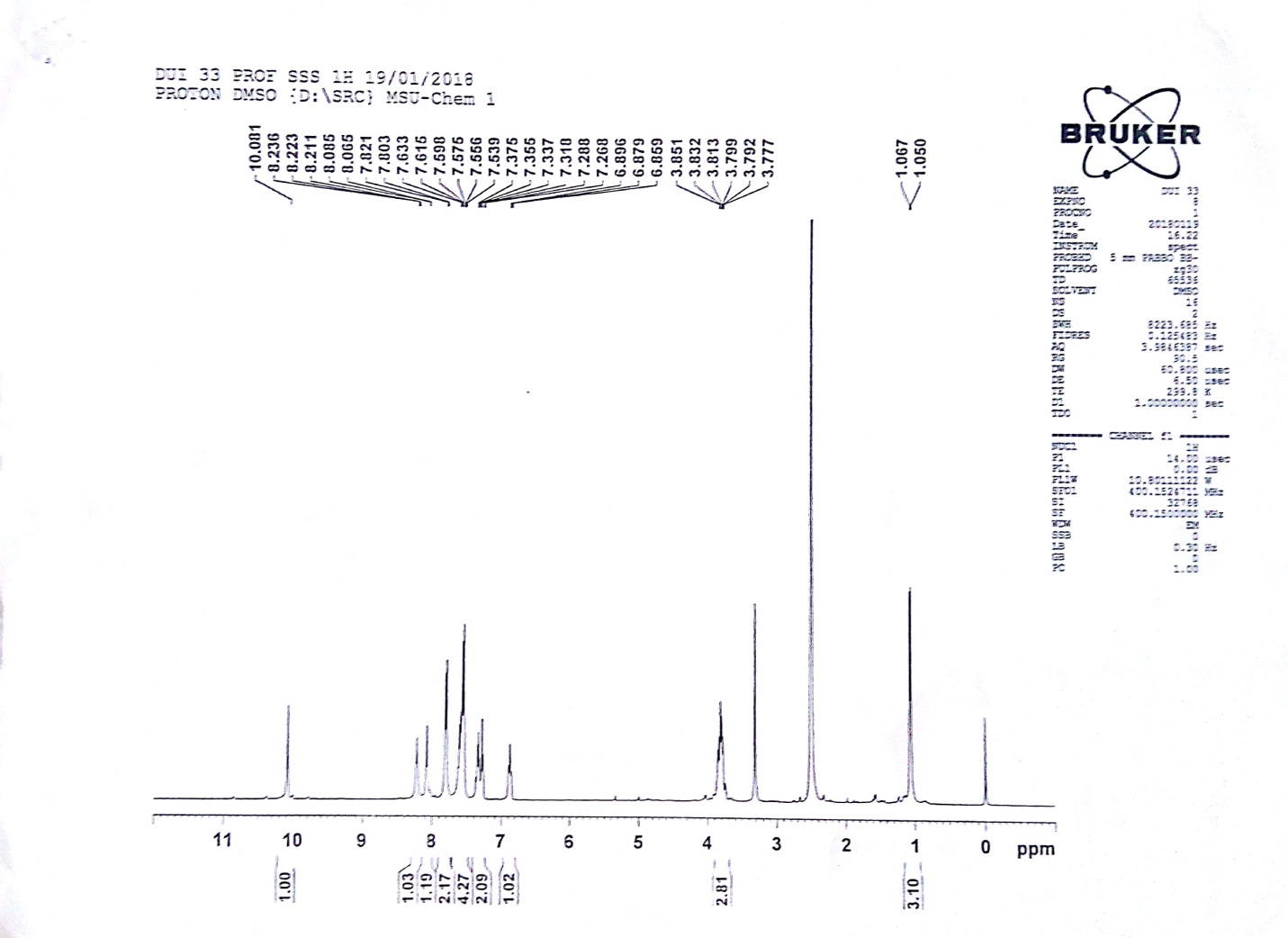


7c

Supplement: Supplemental Material [file IENZ_A_1651313_SM2427.zip › 7c 1H.docx]

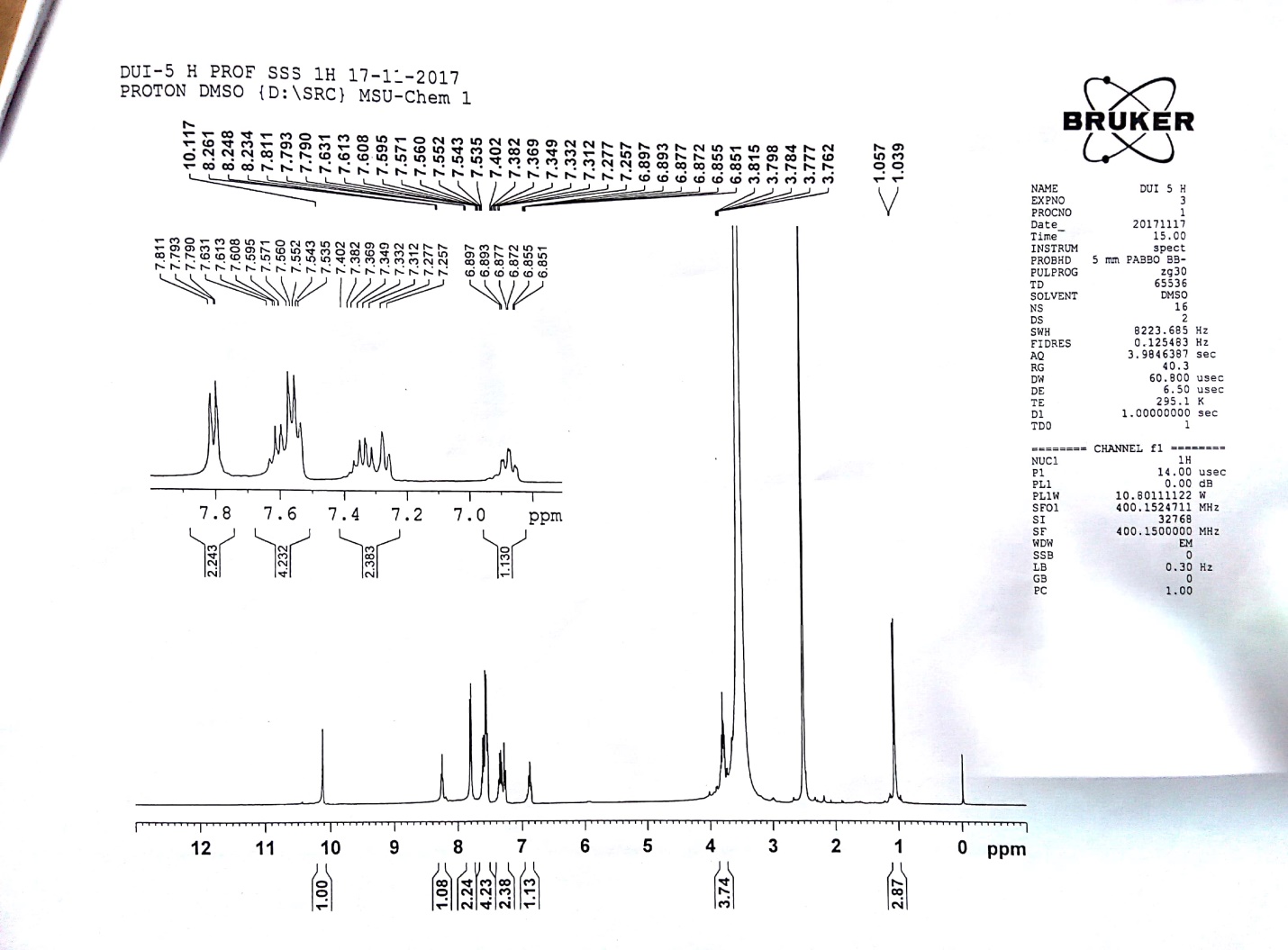


7d

Supplement: Supplemental Material [file IENZ_A_1651313_SM2427.zip › 7d 1H.docx]

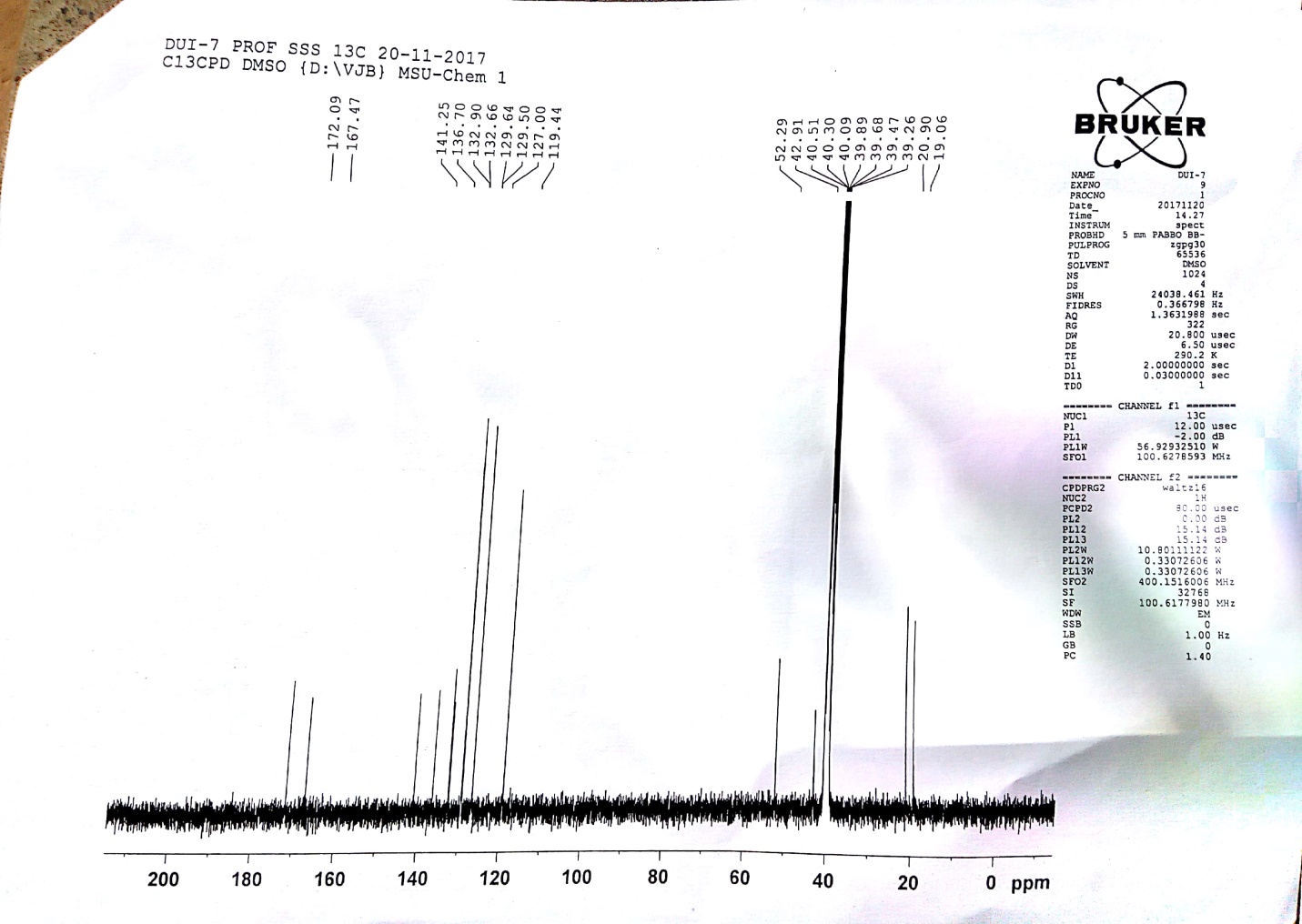


7e

Supplement: Supplemental Material [file IENZ_A_1651313_SM2427.zip › 7e 13C.docx]

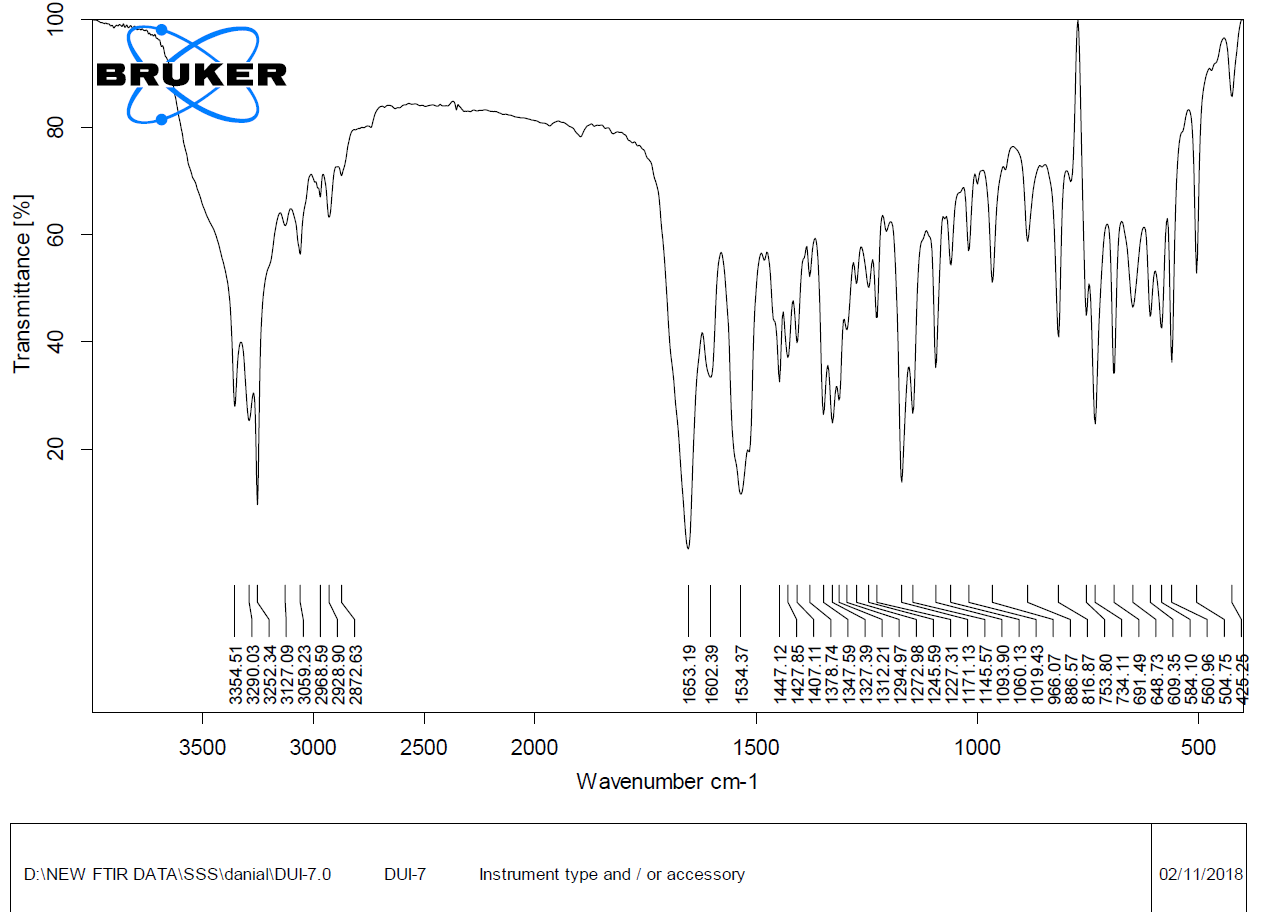


7e

Supplement: Supplemental Material [file IENZ_A_1651313_SM2427.zip › 7e ir.docx]

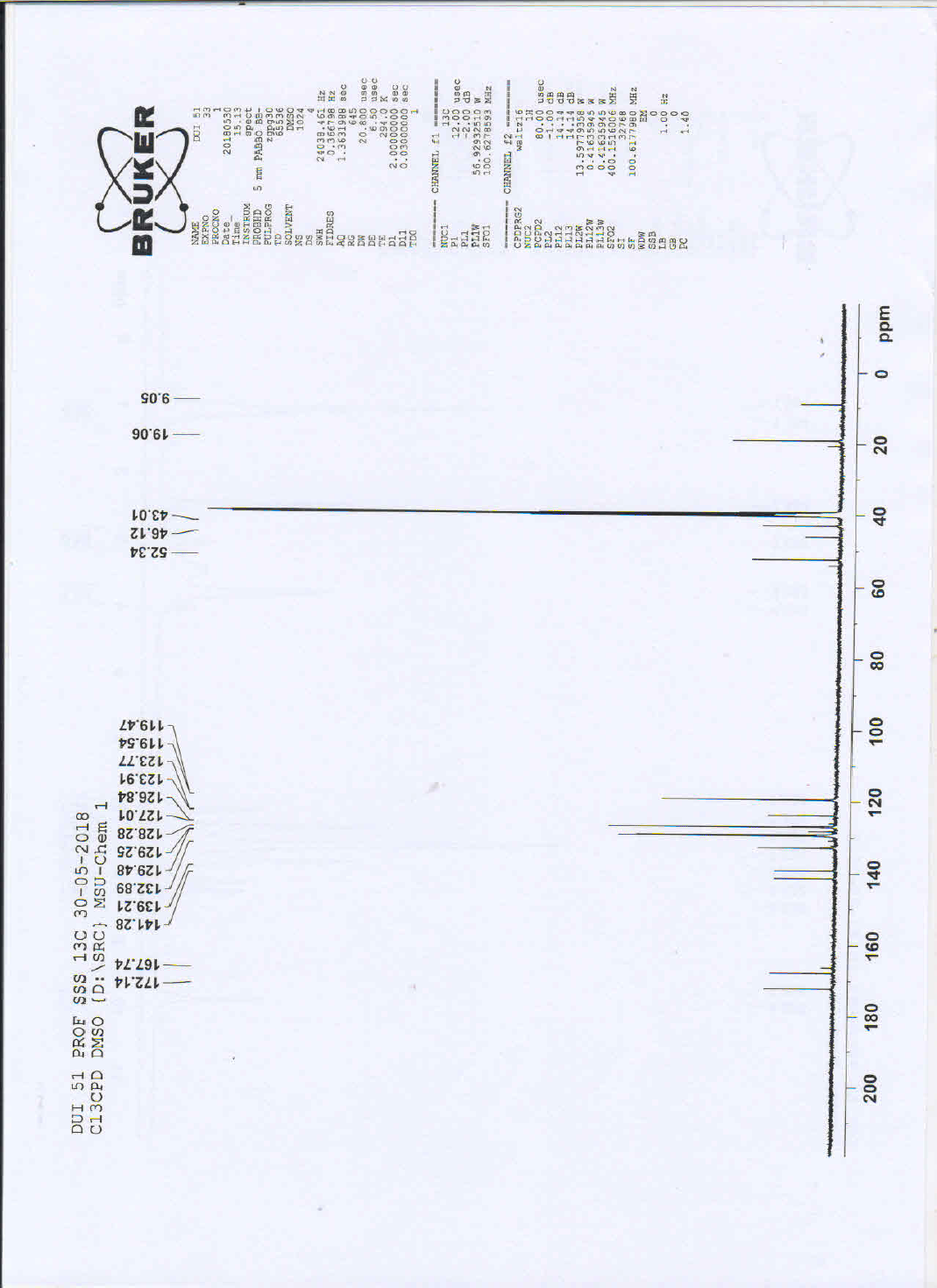


7f

Supplement: Supplemental Material [file IENZ_A_1651313_SM2427.zip › 7f 13C.docx]

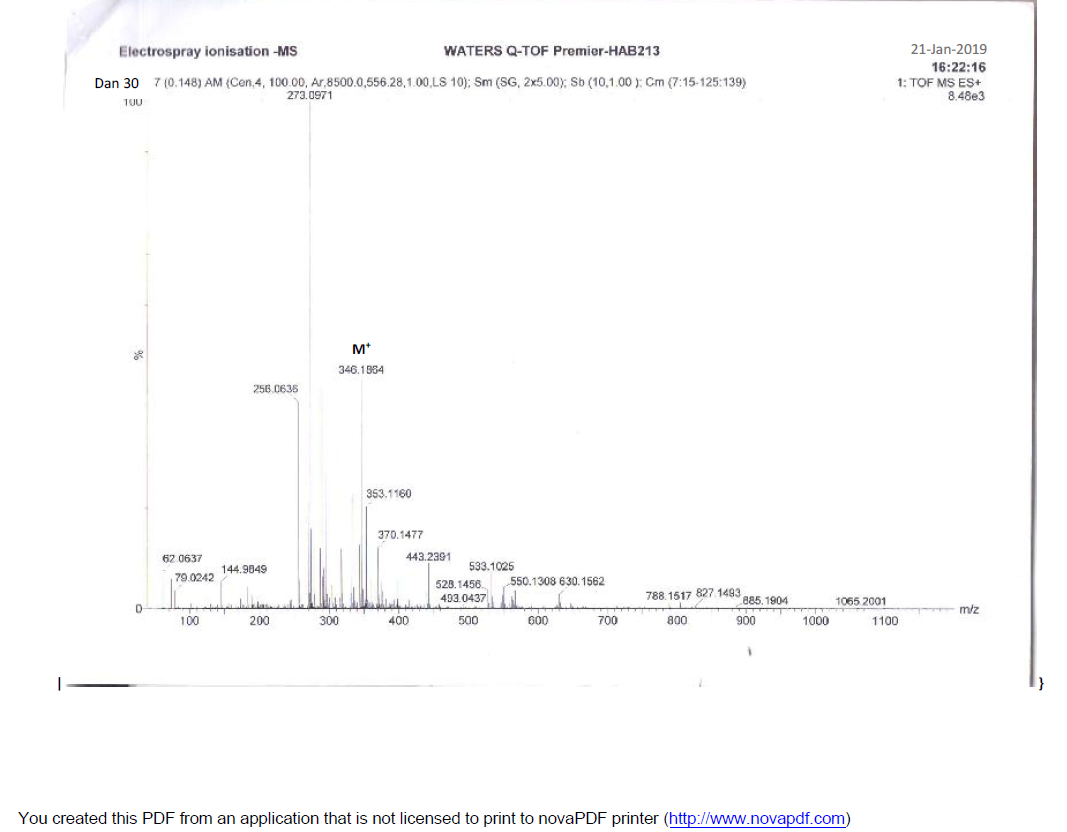


7f

Supplement: Supplemental Material [file IENZ_A_1651313_SM2427.zip › 7f mass.docx]

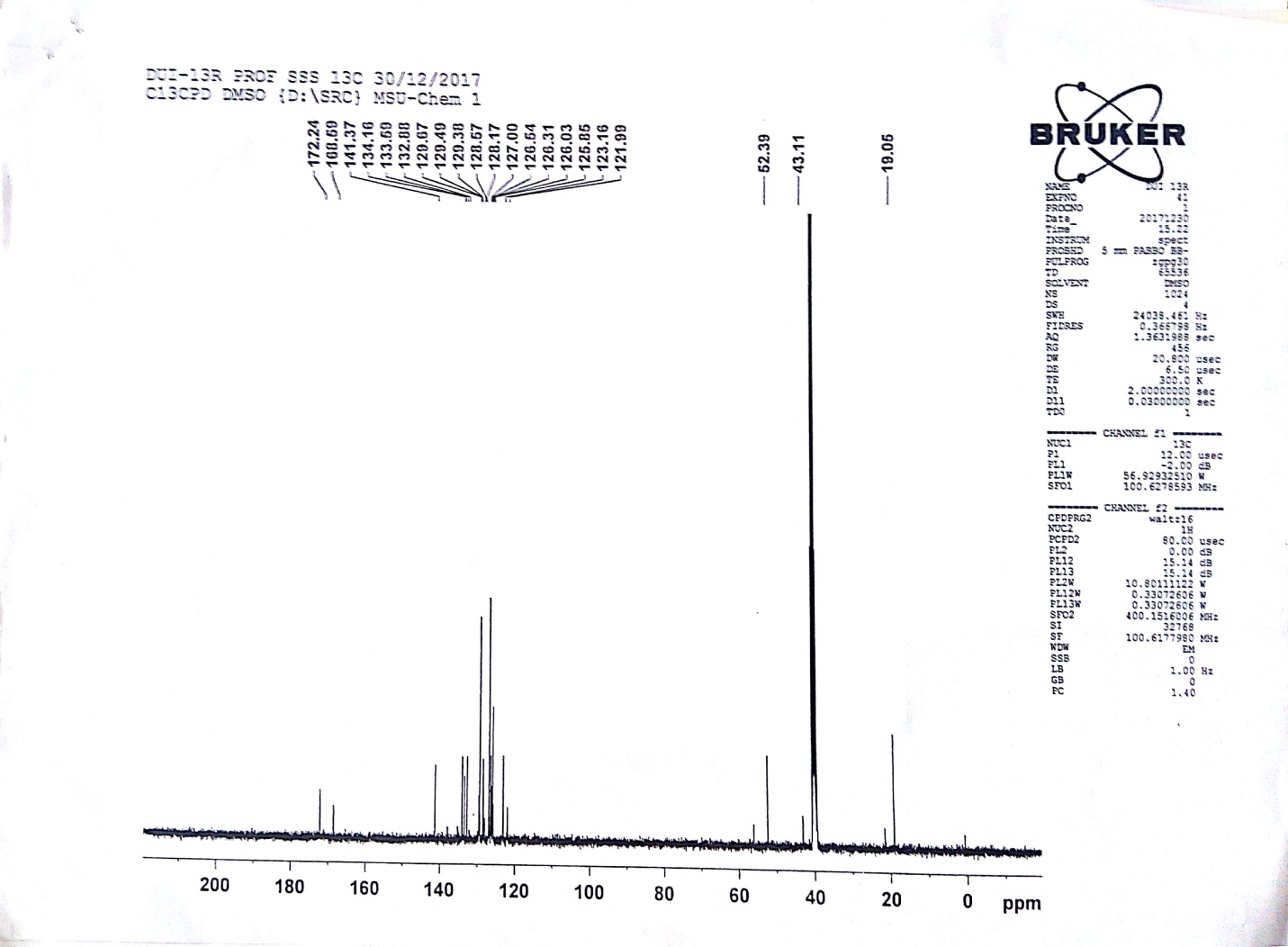


7g

Supplement: Supplemental Material [file IENZ_A_1651313_SM2427.zip › 7g 13C.docx]

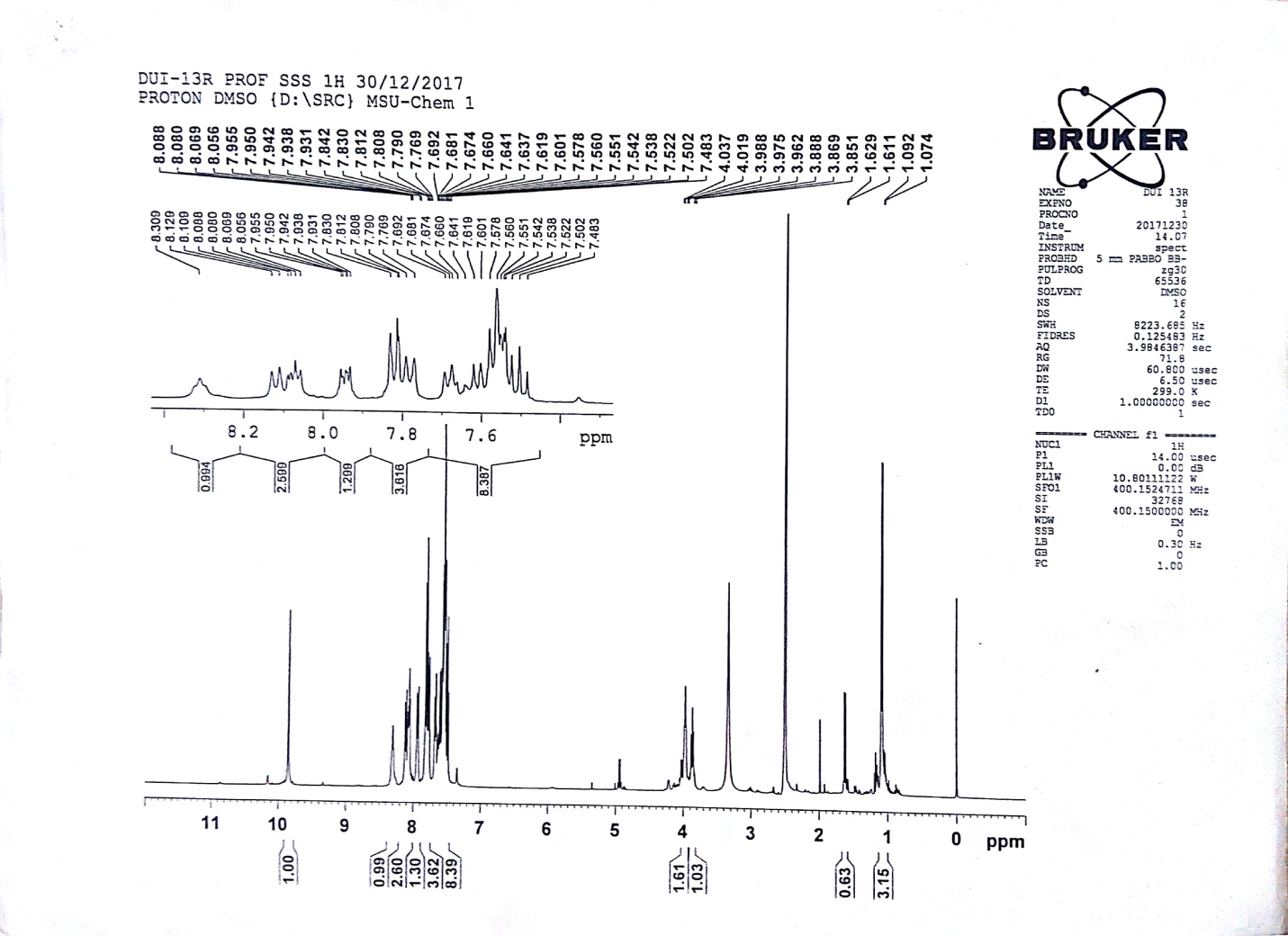


7g

Supplement: Supplemental Material [file IENZ_A_1651313_SM2427.zip › 7g 1H.docx]

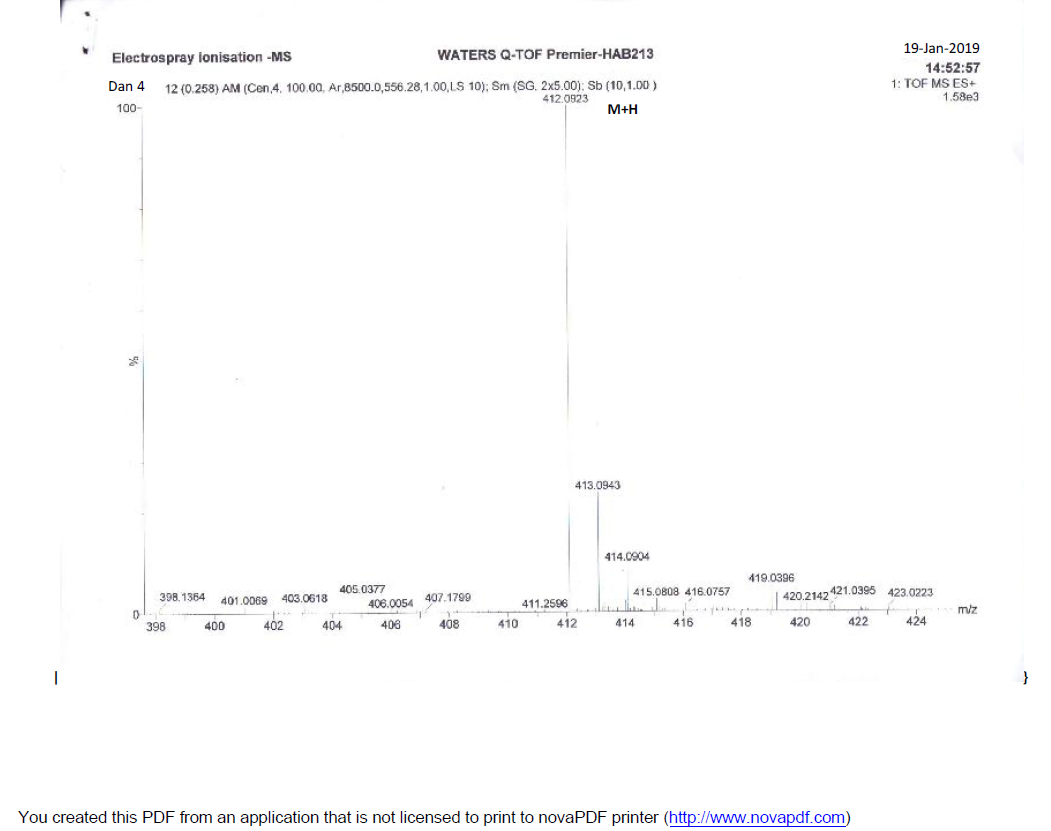


7g

Supplement: Supplemental Material [file IENZ_A_1651313_SM2427.zip › 7g mass.docx]

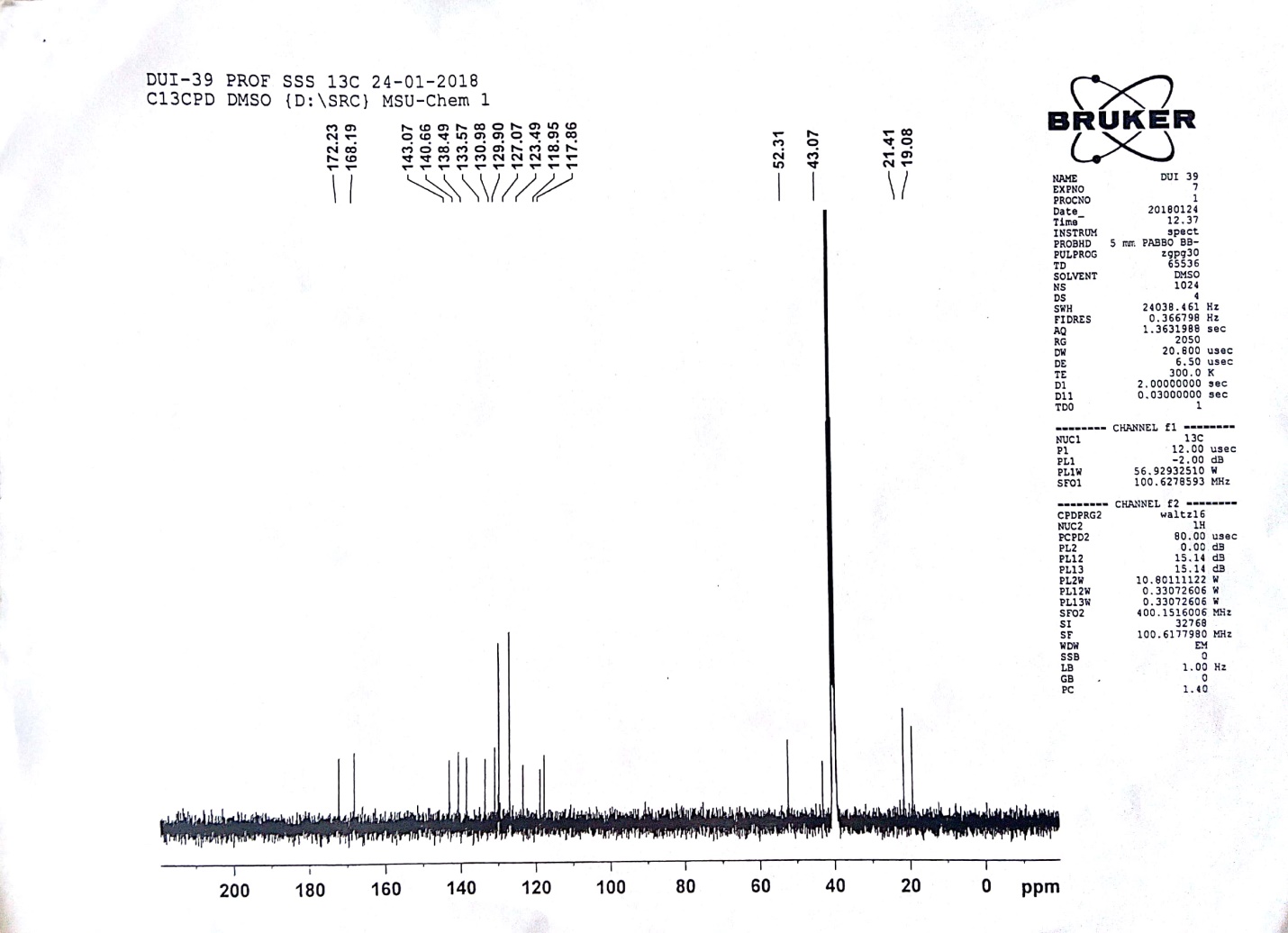


7h

Supplement: Supplemental Material [file IENZ_A_1651313_SM2427.zip › 7h 13C.docx]

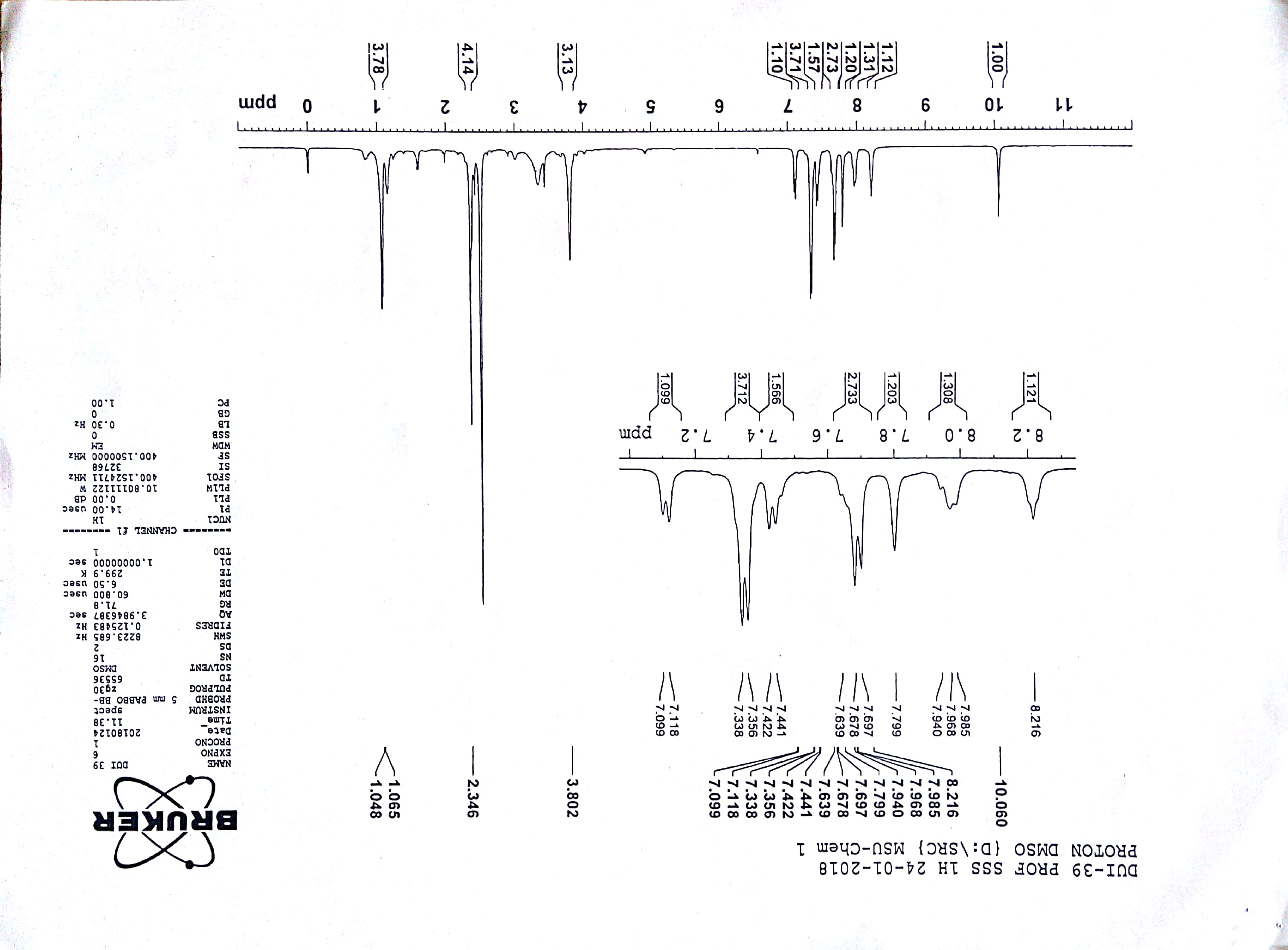


7h

Supplement: Supplemental Material [file IENZ_A_1651313_SM2427.zip › 7h 1H.docx]

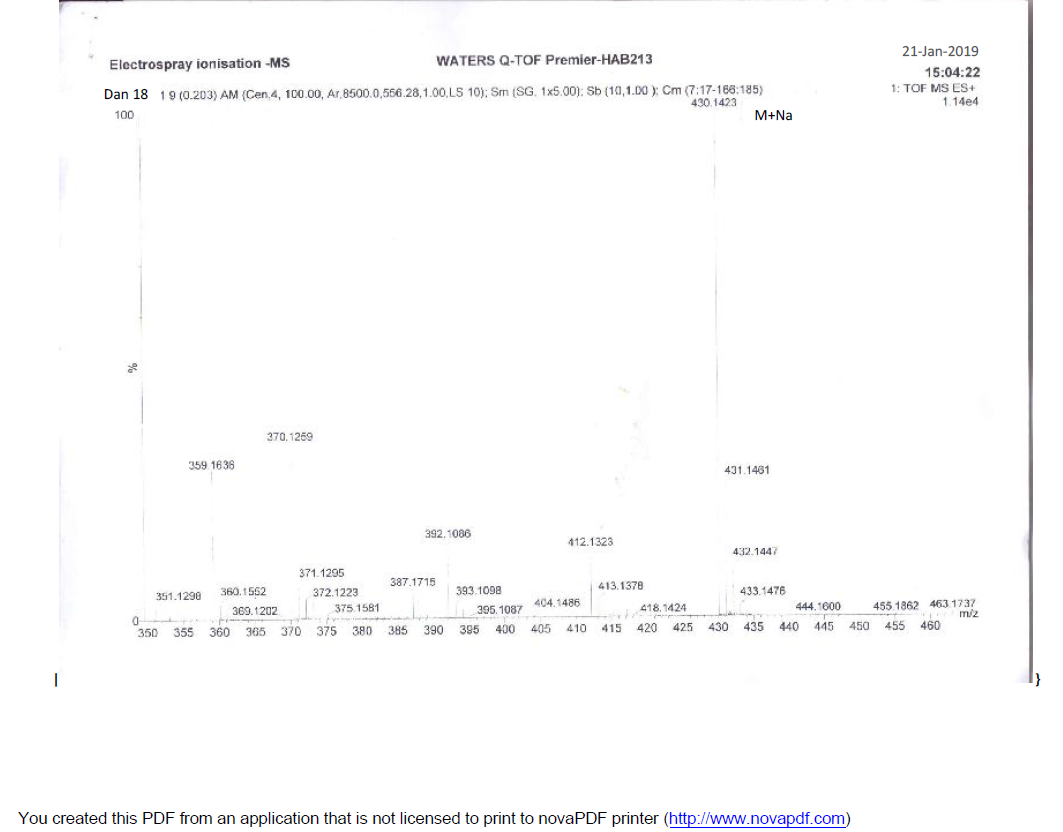


7h

Supplement: Supplemental Material [file IENZ_A_1651313_SM2427.zip › 7h mass.docx]

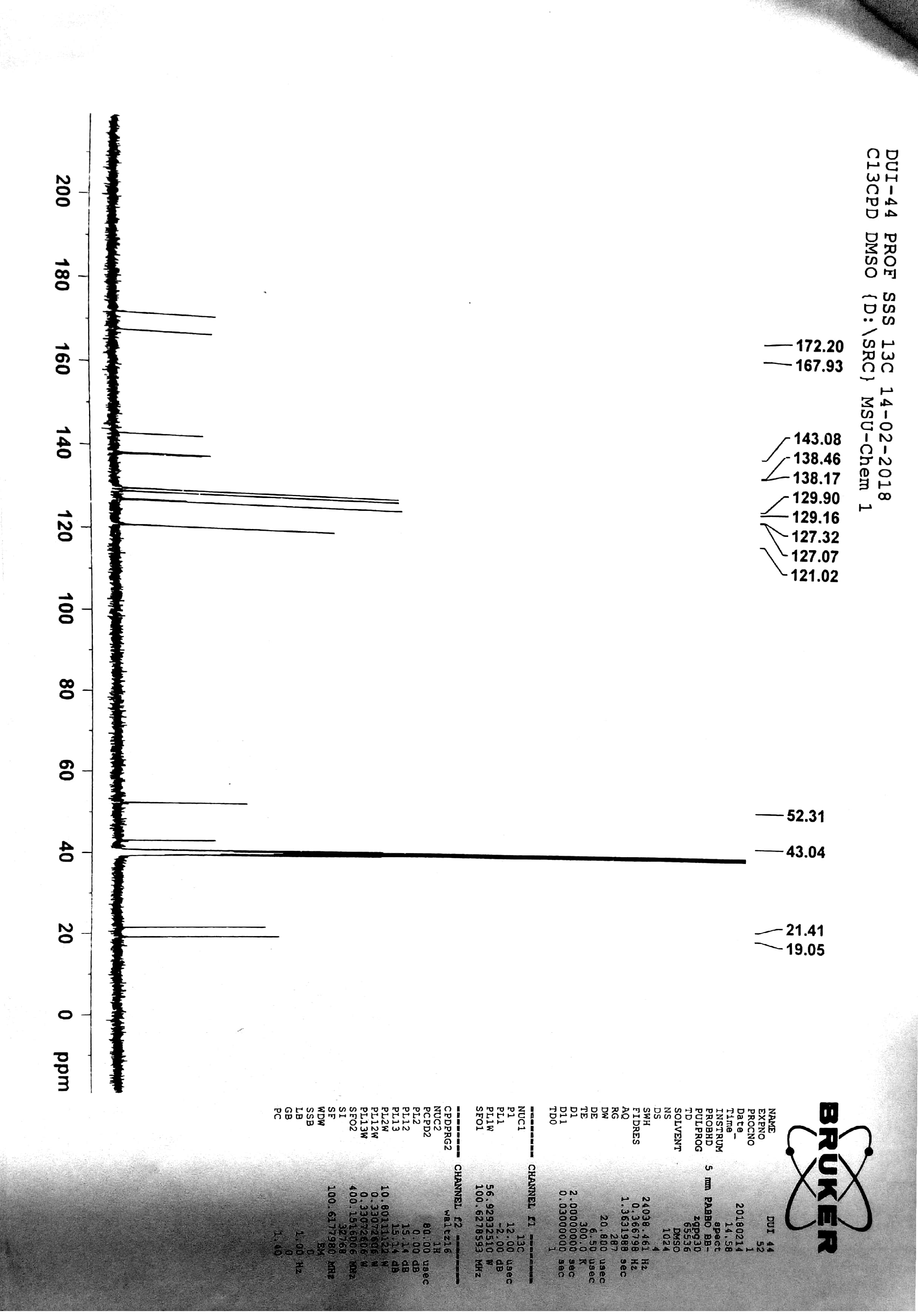


7i

Supplement: Supplemental Material [file IENZ_A_1651313_SM2427.zip › 7i 13C.docx]

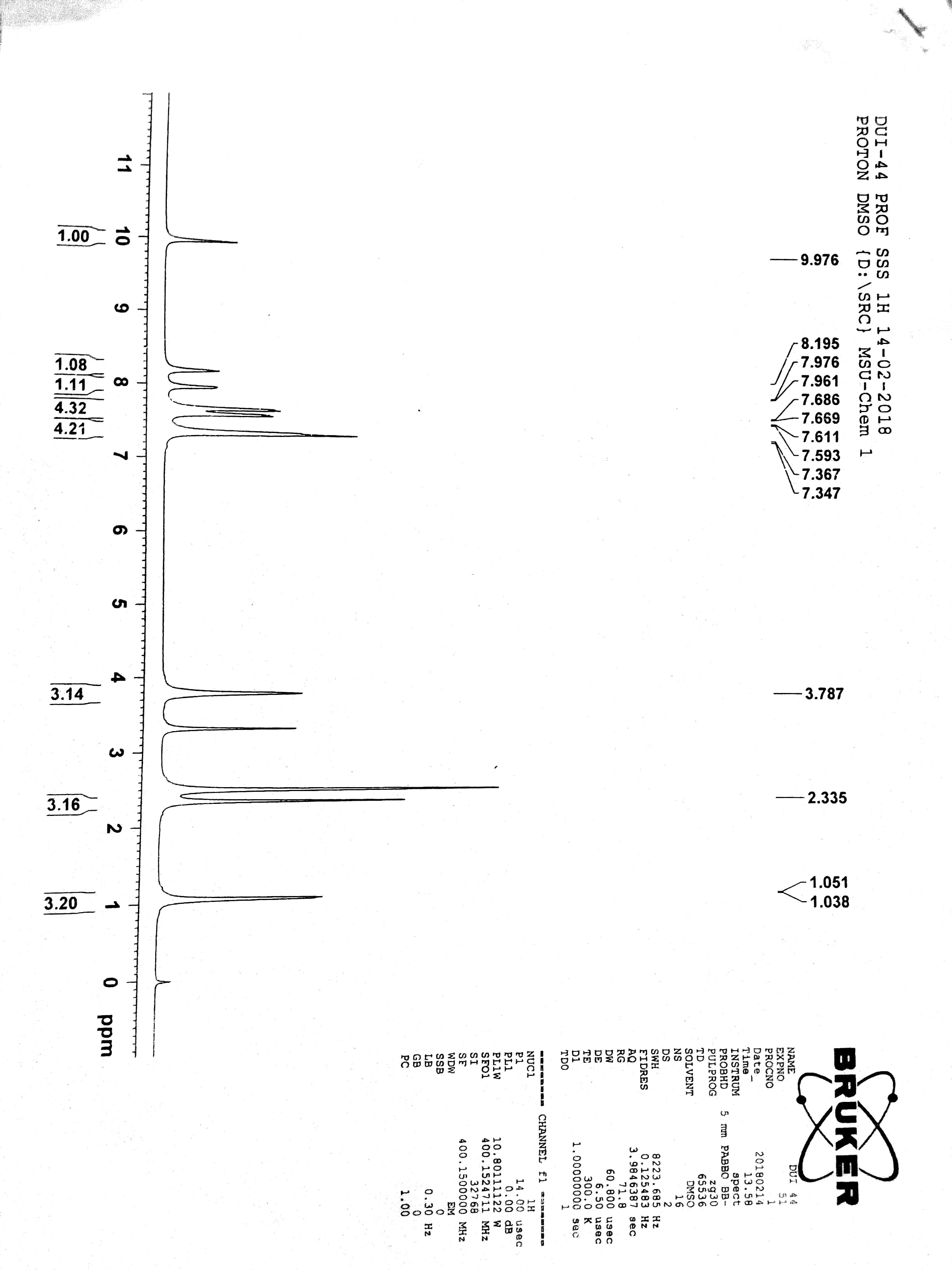


7i

Supplement: Supplemental Material [file IENZ_A_1651313_SM2427.zip › 7i 1H.docx]

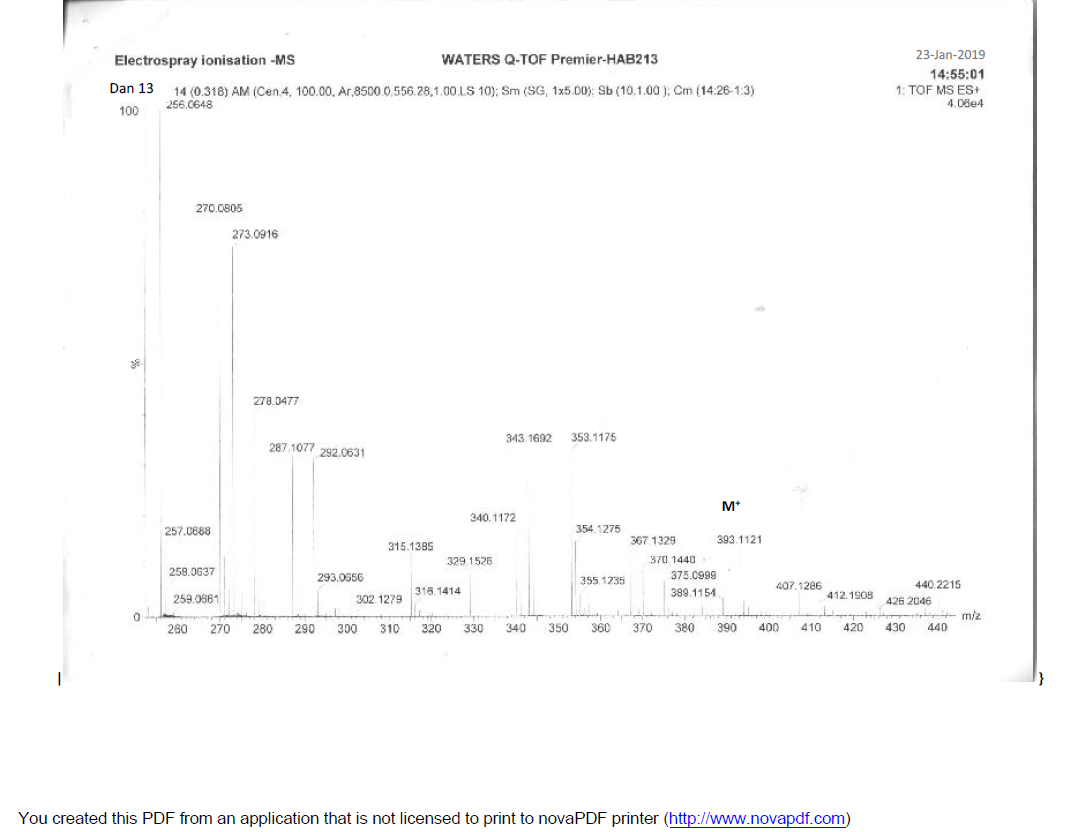


7j

Supplement: Supplemental Material [file IENZ_A_1651313_SM2427.zip › 7j mass.docx]

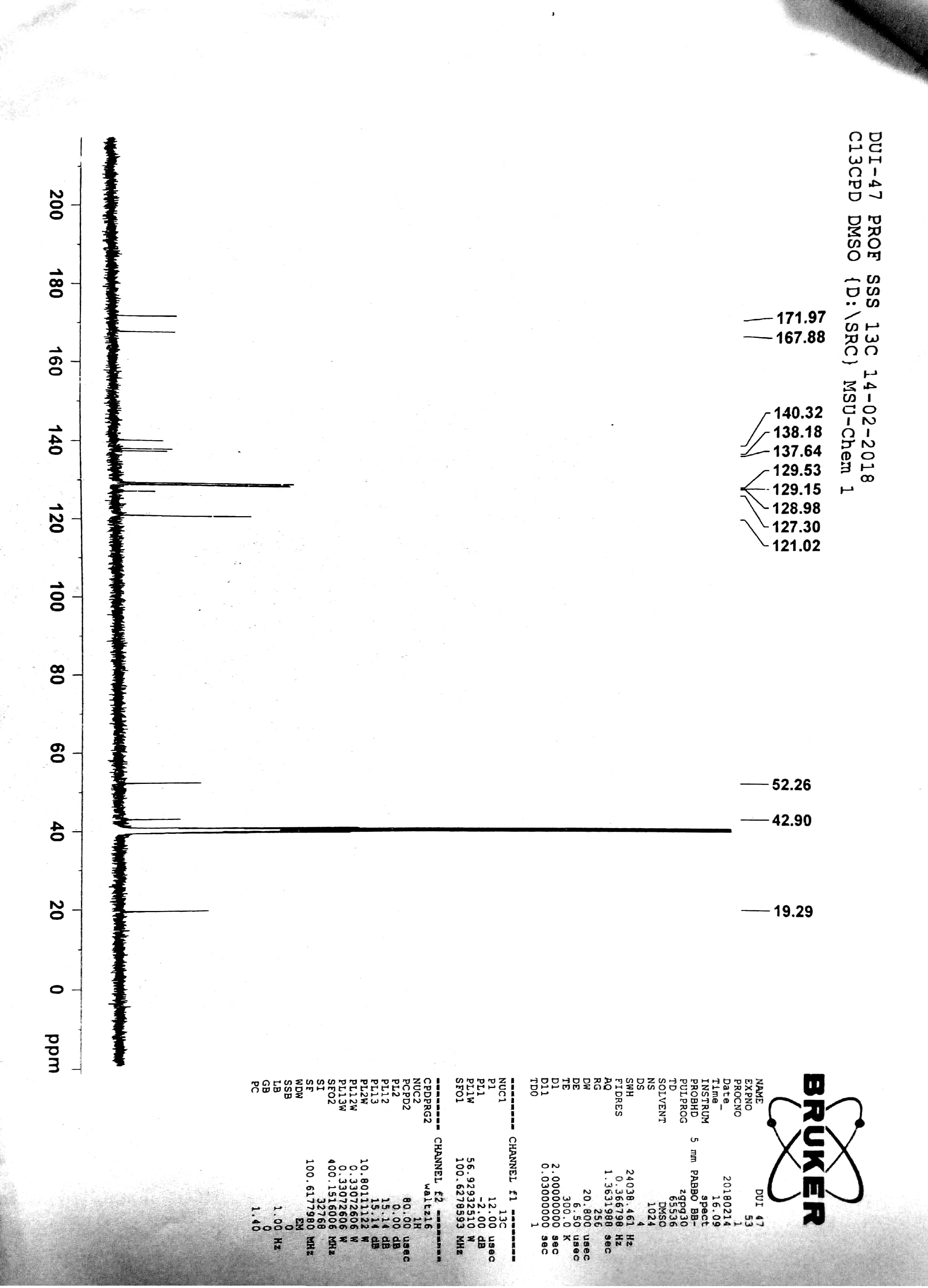


7l

Supplement: Supplemental Material [file IENZ_A_1651313_SM2427.zip › 7l 13C.docx]

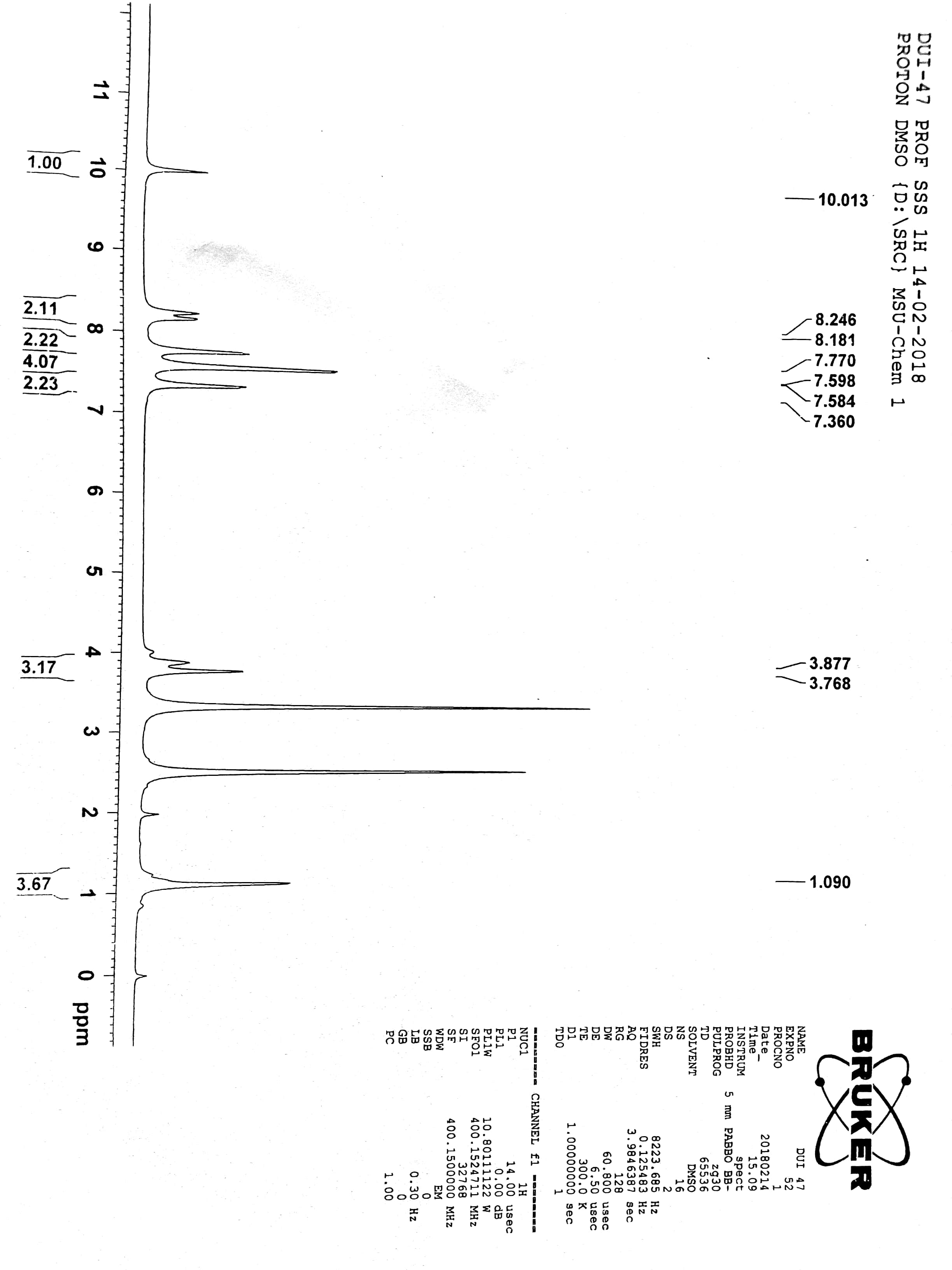


7l

Supplement: Supplemental Material [file IENZ_A_1651313_SM2427.zip › 7l 1H.docx]

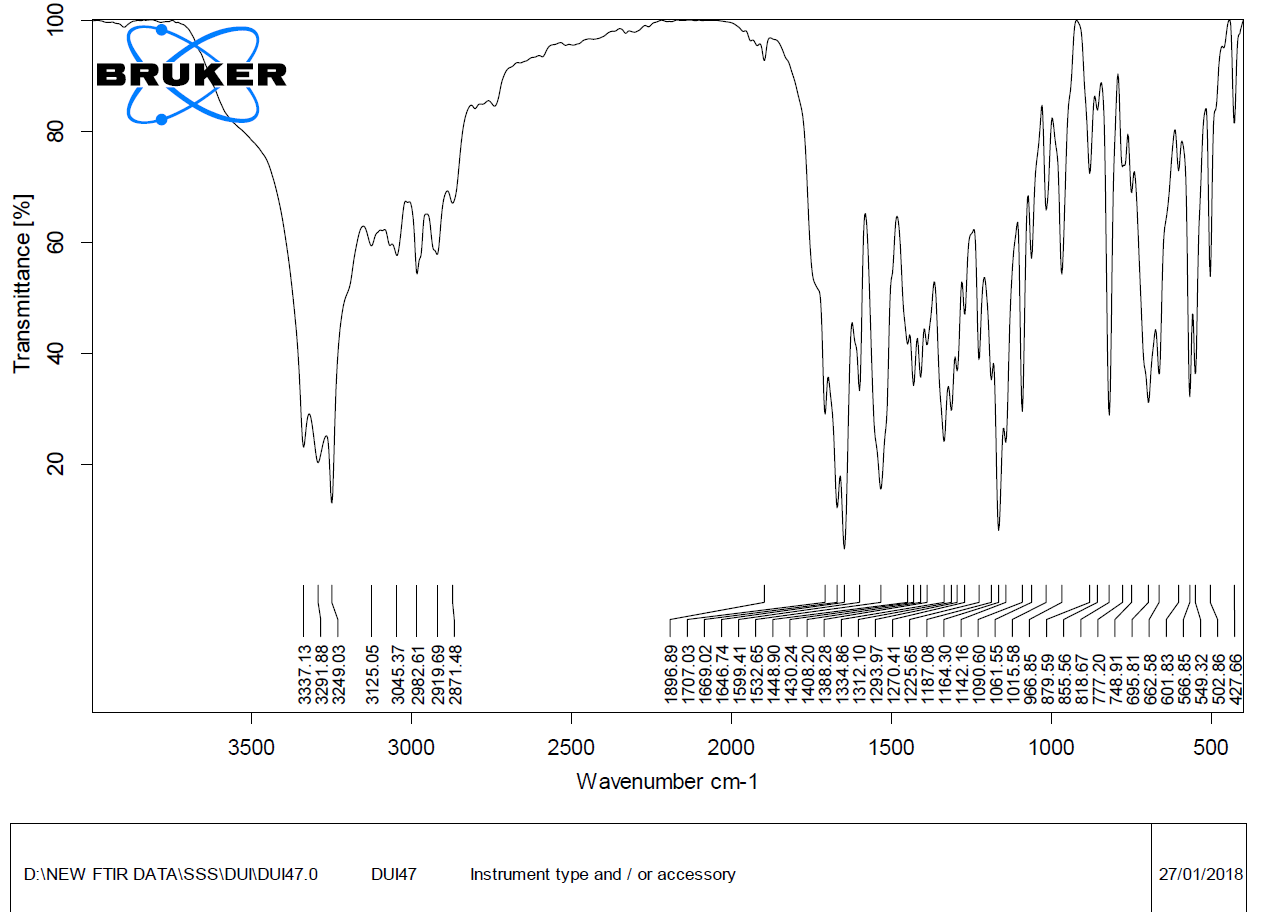


7l

Supplement: Supplemental Material [file IENZ_A_1651313_SM2427.zip › 7l ir.docx]

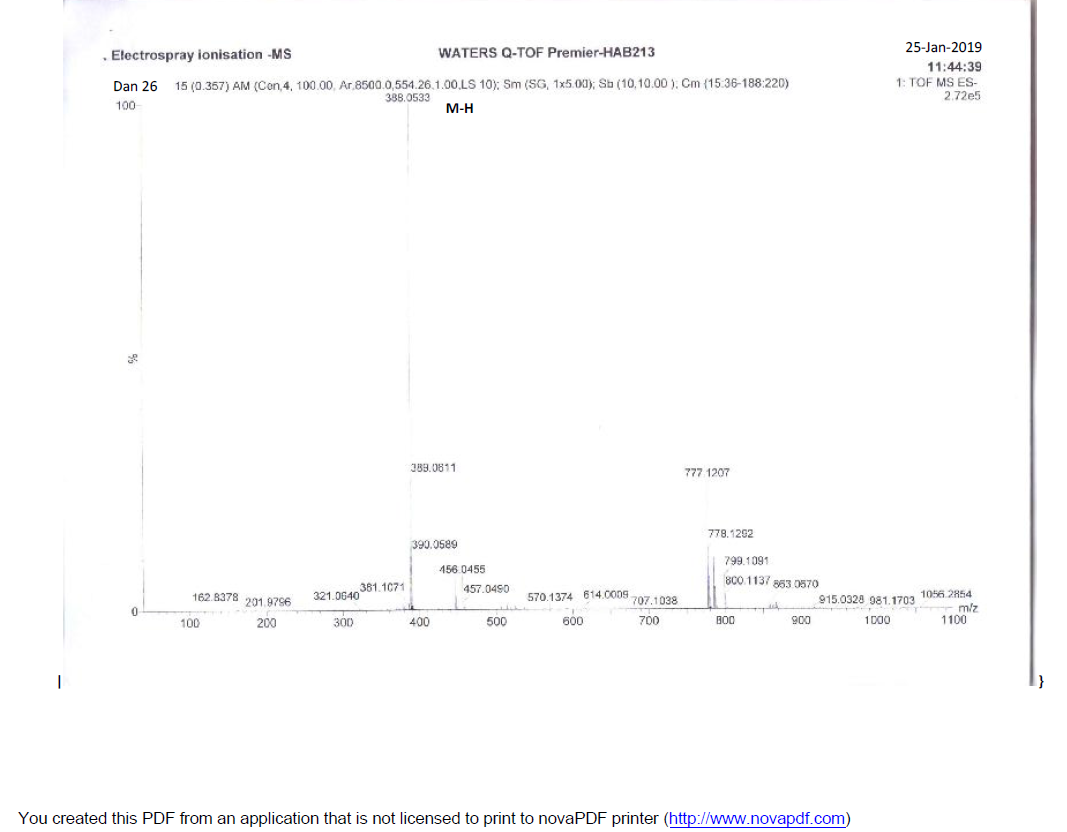


7l

Supplement: Supplemental Material [file IENZ_A_1651313_SM2427.zip › 7L mass.docx]

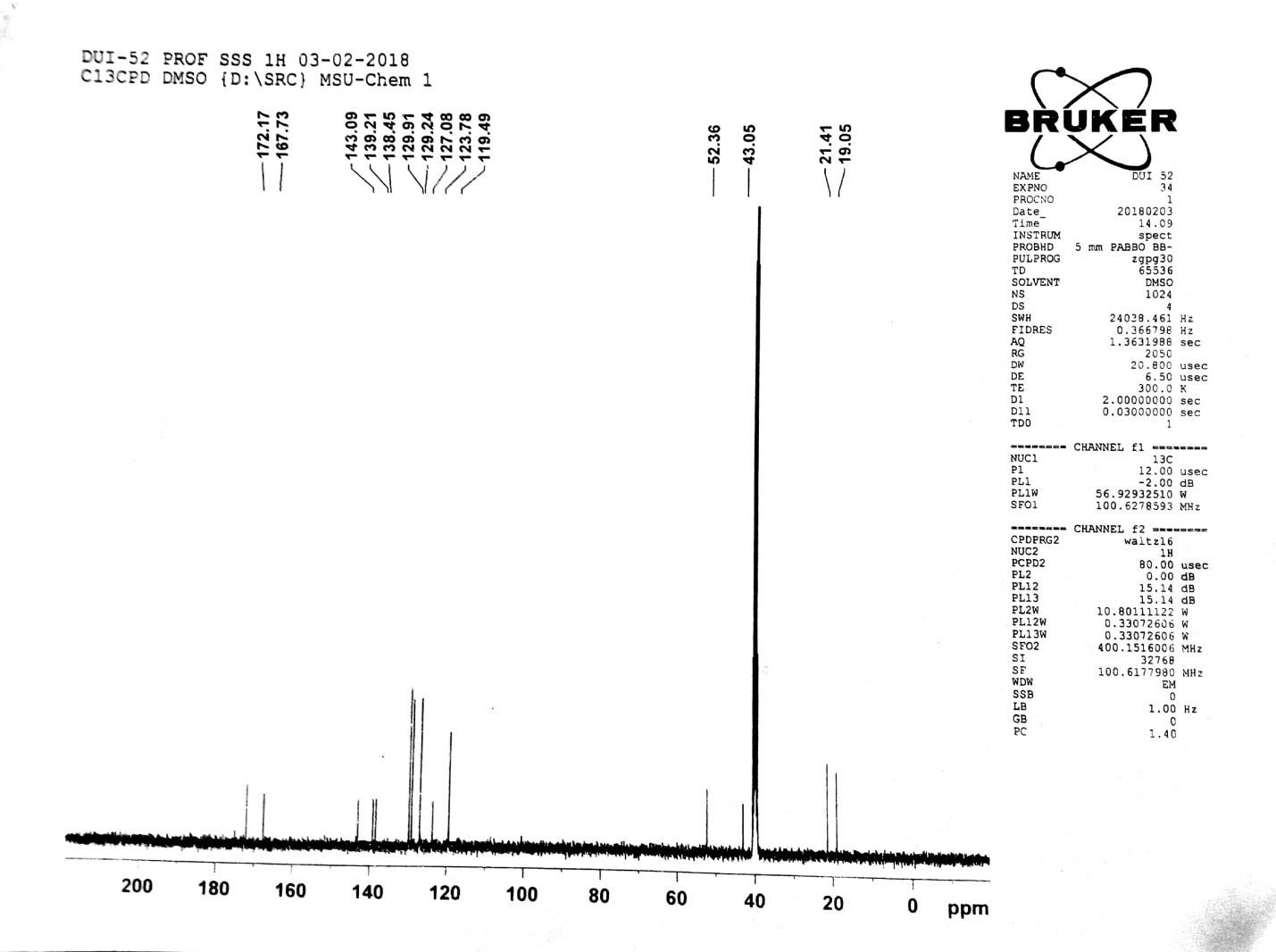


7m

Supplement: Supplemental Material [file IENZ_A_1651313_SM2427.zip › 7m 13C.docx]

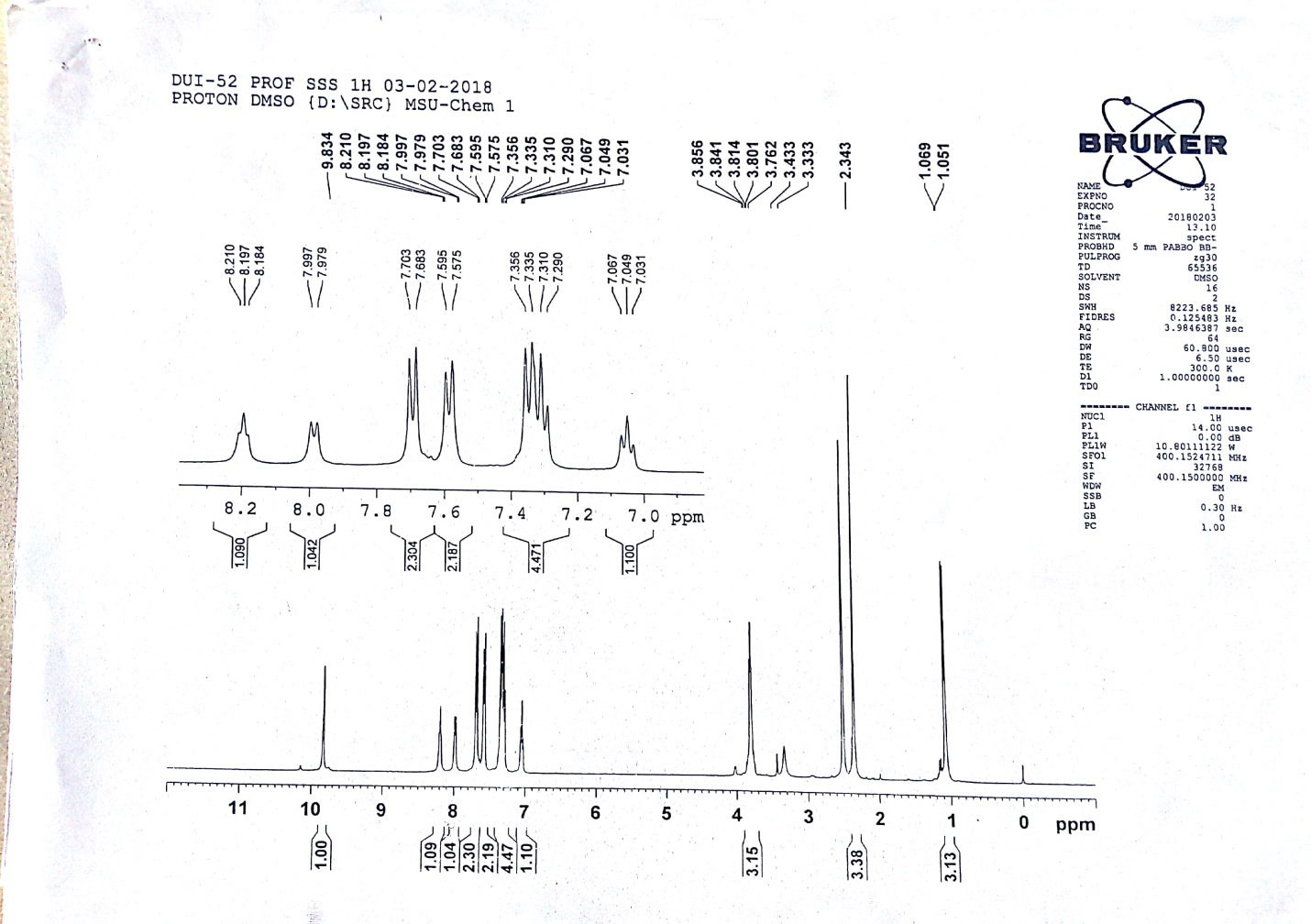


7m

Supplement: Supplemental Material [file IENZ_A_1651313_SM2427.zip › 7m 1H.docx]

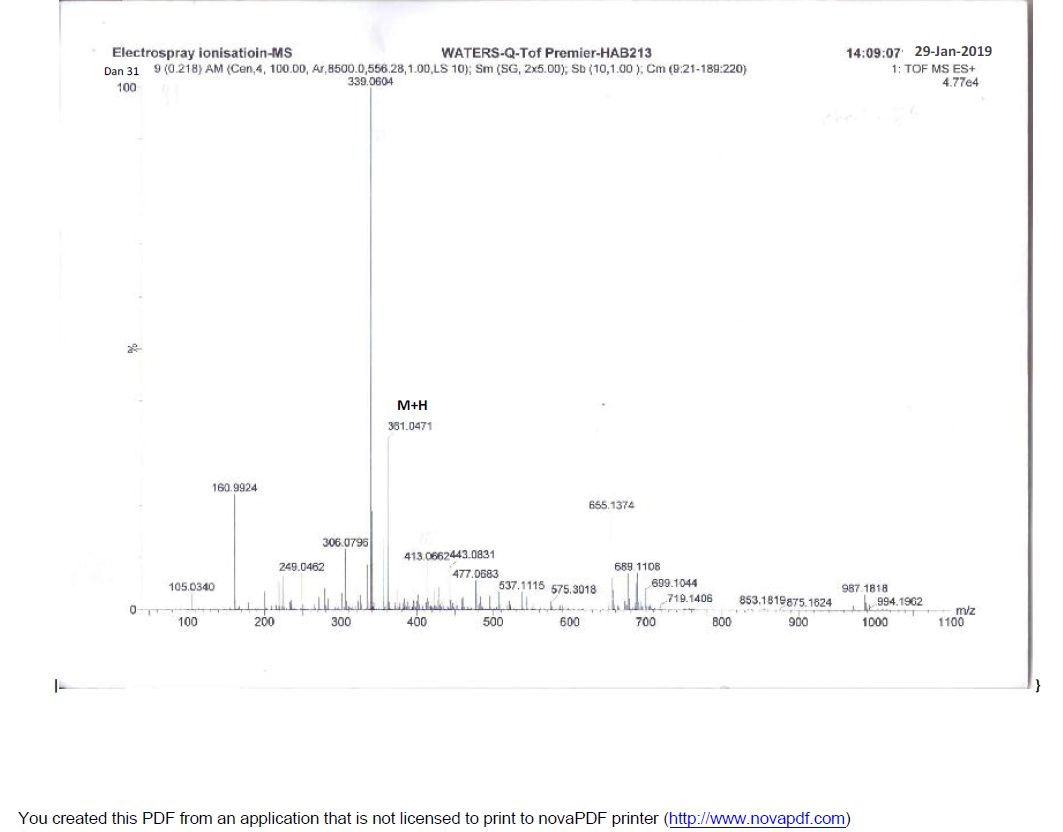


7m

Supplement: Supplemental Material [file IENZ_A_1651313_SM2427.zip › 7m mass.docx]

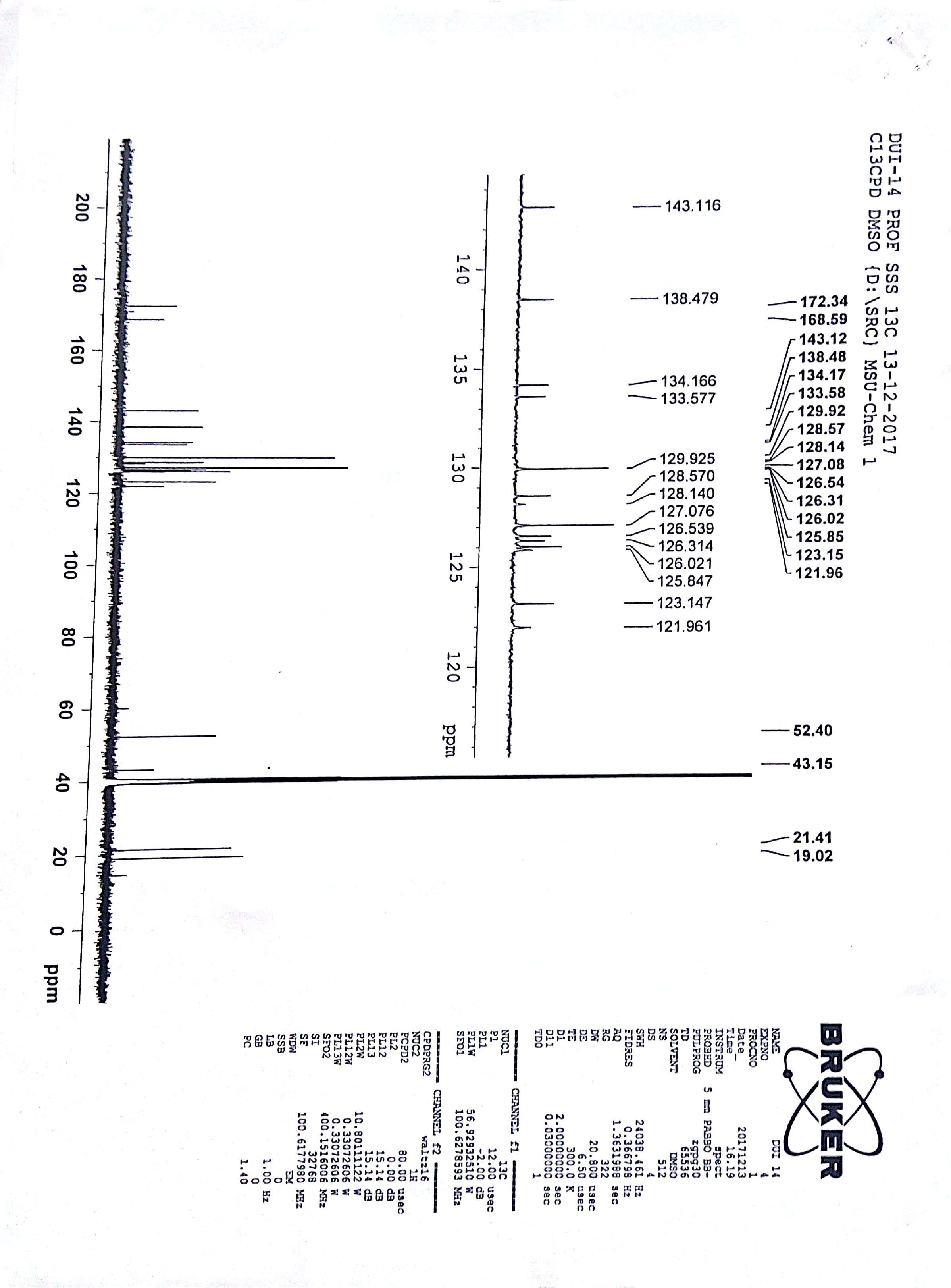


7n

Supplement: Supplemental Material [file IENZ_A_1651313_SM2427.zip › 7n 13C.docx]

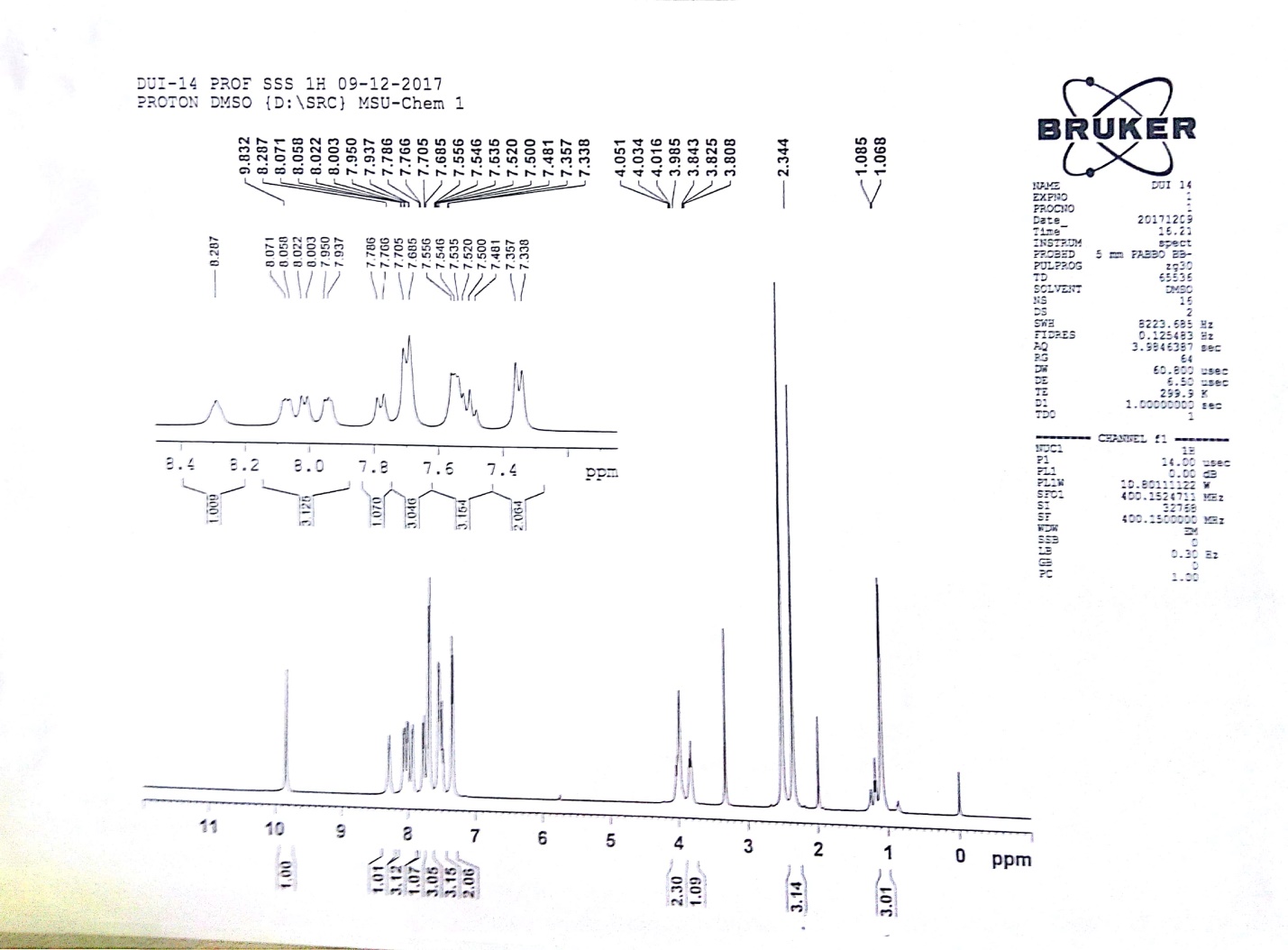


7n

Supplement: Supplemental Material [file IENZ_A_1651313_SM2427.zip › 7n 1H.docx]
